# Fermented dairy product consumption and blood lipid levels in healthy adults: a systematic review

**DOI:** 10.3389/fnut.2025.1651134

**Published:** 2025-09-11

**Authors:** Birsen Yilmaz, Panagiota Alvanoudi, Aggeliki Kalogeropoulou, Dushica Santa, Tuğçe Bulmuş-Tüccar, Anastasios Nikolaou, Isabel Moreno-Indias, Patricia Ruiz-Limon, Carolina Gutiérrez-Repiso, Elaine Hillesheim, Victoria Meslier, Baltasar Mayo, Jeadran Malagon, Christophe Chassard, Smilja Praćer, Guy Vergeres, Fani Th Mantzouridou

**Affiliations:** ^1^Department of Biological Sciences, Tata Institute of Fundamental Research, Hyderabad, India; ^2^Advanced Research Unit on Metabolism, Development and Ageing (ARUMDA), Tata Institute of Fundamental Research, Hyderabad, India; ^3^Laboratory of Food Chemistry and Technology, School of Chemistry, Aristotle University of Thessaloniki, Thessaloniki, Greece; ^4^Faculty of Agricultural Sciences and Food, Ss. Cyril and Methodius University in Skopje, Skopje, North Macedonia; ^5^Department of Nutrition and Dietetics, Yüksek İhtisas University, Ankara, Türkiye; ^6^Department of Molecular Biology & Genetics, Democritus University of Thrace, Alexandroupolis, Greece; ^7^Instituto de Investigación Biomédica de Málaga y Plataforma en Nanomedicina-IBIMA Plataforma BIONAND, Málaga, Spain; ^8^Department of Endocrinology and Nutrition Virgen de la Victoria University Hospital, Málaga, Spain; ^9^Centro de Investigación Biomédica en Red de la Fisiopatología de la Obesidad y Nutrición (CIBEROBN), Instituto de Salud Carlos III, Madrid, Spain; ^10^Agroscope, Liebefeld, Bern, Switzerland; ^11^Université Paris-Saclay, INRAE, MetaGenoPolis, Jouy-en-Josas, France; ^12^Instituto de Productos Lácteos de Asturias (IPLA-CSIC), Oviedo, Spain; ^13^Observatorio Nacional de Salud, Instituto Nacional de Salud, Bogotá, Colombia; ^14^INRAE, UMR 1019, UNH, CRNH Auvergne, Clermont-Ferrand, France; ^15^Clermont Université, Université d'Auvergne, Unité de Nutrition Humaine, Clermont-Ferrand, France; ^16^Institute for Biological Research Siniša Stanković, National Institute of the Republic of Serbia, University of Belgrade, Belgrade, Serbia

**Keywords:** fermented dairy, yogurt, kefir, cheese, cholesterol, triglycerides, metabolic markers, cardiovascular diseases

## Abstract

Cardiovascular diseases remain the leading cause of global mortality, with diet recognized as a key factor influencing cardiovascular risk biomarkers such as blood lipids. Emerging evidence suggests fermented dairy products may offer cardioprotective benefits via fermentation-derived bioactive metabolites. This systematic review, conducted within COST Action CA20218 Promoting Innovation of ferMENTed fOods (PIMENTO), evaluated the relationship between consumption of conventional fermented dairy products and blood lipid levels and cardiovascular diseases in healthy adults. Data were qualitatively summarized and synthesized narratively, following the European Food Safety Authority’s (EFSA) scientific guidance for health claim applications, including food characterization, bioavailability of relevant compounds, mechanisms of action, and safety. Sixty-eight studies were included: 14 controlled interventions (PICO), 37 non-controlled interventions (PIO), and 17 observational studies. Findings from intervention studies were largely inconsistent, with most trials reporting no significant changes in lipid markers following fermented dairy intake. However, a few studies reported modest reductions in total cholesterol and LDL-c or improved LDL/HDL ratios, particularly with yoghurt and kefir. Observational studies also yielded mixed and inconclusive results. Overall, study quality, result consistency, and mechanistic evidence were deemed “neither convincing nor sufficient” per EFSA criteria. Key limitations included high risk of bias, heterogeneous designs, inadequate product characterization, and limited mechanistic data. More rigorous, well-controlled human studies with appropriate comparators are needed to clarify whether conventional fermented dairy products have any lipid-lowering effects.

**Systematic review registration:**

osf.io/h2mbe/

## 1 Introduction

Cardiovascular diseases (CVDs) are a group of conditions that primarily affect the heart and blood vessels (1). They are often the consequences of chronic health conditions such as dyslipidemia, hypertension, insulin resistance, chronic inflammation and atherosclerosis (2, 3). CVDs remain the leading cause of mortality worldwide, accounting for 32% of all deaths, with the majority (nearly 85%) attributed to heart attacks and strokes (1, 2). The global CVD-related death toll has grown substantially in recent decades (4, 5), with over three-quarters of these deaths occurring in low-and middle-income countries (1). This increase may be attributed to several factors, including aging populations, sedentary lifestyles, rising obesity rates, and the growing prevalence of metabolic disorders, which often manifest as the very conditions that drive CVD progression. Notably, well-established biomarkers such as total cholesterol, low-density lipoprotein cholesterol (LDL-c), high-density lipoprotein cholesterol (HDL-c), and triglycerides are recognized risk factors for CVD (6), and are strongly influenced by an individual’s dietary habits.

Modifiable lifestyle factors, especially diet, are known to play a crucial role in both the development and mitigation of cardiovascular risk, affecting individuals as well as entire populations (7). Dairy products, both fermented and non-fermented, constitute an important component of many diets worldwide and have a long history of manufacture, consumption, and cultural significance. Recently, increasing attention has been drawn to the potential effects of dairy consumption on CVDs (8), with several cohort studies indicating that higher or more frequent intake may be associated with a reduced risk of CVDs (9–11). Beyond their well-known abundance of essential minerals and vitamins, the beneficial effects of dairy may also be attributed to bioactive peptides and fermentation-derived metabolites, which are believed to exhibit enhanced bioavailability in fermented products (2).

Building on this evidence, research has increasingly focused on fermented dairy products, particularly yogurt and traditionally aged cheeses, and their distinct cardioprotective properties. Although broader dairy research has yielded mixed findings (12, 13), emerging data from both observational and interventional studies suggest that regular consumption of fermented dairy may improve lipid levels and endothelial function, regulate blood pressure, and reduce stroke risk (14, 15). In parallel, advances in omics technologies have helped identify specific bioactive peptides and fermentation-derived metabolites that may modulate key cardiovascular pathways, genetic factors, and gut microbiome composition (16, 17). Collectively, these findings highlight the need to investigate whether and how fermented dairy products should be differentiated from non-fermented forms in dietary guidance, including the specification of dairy type and fermentation methods, while accounting for population- or even individual-level metabolic profiles when evaluating CVD outcomes.

Motivated by the emerging evidence, this systematic review aims to critically evaluate the relationship between the consumption of fermented dairy products and blood lipid levels and cardiovascular diseases in healthy adults. The research question guiding this review is: *Does consumption of fermented dairy products impact blood lipids in healthy adults?* To address this, a systematic appraisal of evidence from intervention and observational studies is carried out. In addition, we conduct a targeted non-systematic review of the product characteristics, including the presence and bioavailability of fermentation-derived bioactive compounds, such as peptides and short-chain fatty acids, that may contribute to the reported effects and their underlying mechanisms of action, and modulate lipid metabolism and inflammatory pathways. By applying the EFSA guidelines for health claims, incorporating food characterization, bioavailability, mechanisms of action, and safety, this review offers a novel and structured framework for evaluating the quality and certainty of evidence, advancing current approaches in the field of nutrition research. Conducted as part of the COST Action CA20218 Promoting Innovation of ferMENTed fOods (PIMENTO) initiative on the health properties of fermented foods (18), this review aims to contribute to the broader understanding of both clinical and mechanistic evidence. The findings aim to identify research gaps and clarify how fermented dairy products may mitigate CVD risk via modulation of blood lipids, thereby informing future dietary recommendations and public health strategies.

## 2 Methods

This review was carried out by subgroup E5 of PIMENTO WG3 (18), comprising ten researchers and co-led by FM and BY. The work of the subgroup was overseen by WG3 co-leads GV and SP, with internal review support provided by the E6 subgroup co-leads BM, JM, and the late Prof. Jyoti Prakash Tamang.

### 2.1 Systematic review of human studies

#### 2.1.1 Study protocol

This systematic review was conducted following the methodological standards of the Cochrane Handbook Systematic Reviews of Interventions (19) and adhered to the PRISMA 2020 statement (20) to ensure transparent and comprehensive reporting. The planning, coordination, iterative updates, and evidence synthesis followed the structured approach proposed by Muka et al. (21) and EFSA guidance (22), with adaptations based on the PIMENTO Study Protocol. The protocol is publicly available on Open Science Framework (DOI: 10.17605/OSF.IO/H2MBE).

#### 2.1.2 Literature search

A comprehensive literature search was conducted to identify human studies investigating the relationship between fermented dairy product consumption and blood lipid levels. The electronic databases (MEDLINE, Scopus, and Cochrane Library) were searched from inception to December 2024. The search strategy was based on the generic search strings developed by the Library of the University of Zurich for the PIMENTO WG3, but adapted to focus specifically on blood lipids and cardiovascular disease outcomes (Supplementary Table S1). Search terms included the combinations of keywords related to fermented dairy products (e.g., yogurt/yogurt, kefir, fermented milk, cheese), lipids (e.g., total cholesterol, triglycerides, LDL-c, HDL-c), cardiovascular diseases (e.g., myocardial infarction, stroke), and study design. Additional relevant studies were identified through screening of systematic and narrative reviews and trial registers. Intervention studies, observational studies, cohort studies, cross-sectional studies, and case–control studies in humans were considered for evaluation, whereas animal and *in vitro* studies were excluded.

#### 2.1.3 Study selection criteria

All references retrieved through the search strategy were uploaded to CADIMA, an evidence synthesis tool (23) where duplicates were automatically removed. Study selection was conducted in two stages which were title and abstract followed by full-text screening. Both stages were performed independently by at least two reviewers. Disagreements were resolved through discussion and consensus or, if necessary, by consulting a third reviewer. Predefined eligibility criteria (Table 1) were applied based on the PICOS framework (Population, Intervention, Comparator, Outcome, and Study Design). Studies that examined conventional fermented dairy products, such as yogurt fermented with classical starter cultures (*Lactobacillus delbrueckii* subsp. *bulgaricus* and *Streptococcus thermophilus*), were included. Enriched versions were considered as products fortified with additional bioactive compounds (e.g., plant sterols) and/or supplemented with probiotic strains after the fermentation process. The latter were excluded, given that their effects could be the result of external fortification and not intrinsic to the fermentation process. In addition to the intervention studies that met PICO criteria, we also included intervention studies that followed a PIO structure, i.e., studies without a control group or where the comparator was deemed unsuitable for the objective of this review. For example, controlled trials comparing the consumption of conventional fermented dairy products with versions enriched with added nutrients or bioactive compounds (e.g., fiber, probiotics, plant sterols) were included under the PIO criteria. In these cases, the group consuming the conventional fermented dairy product group was considered the intervention of interest, while the enriched product group was not relevant for assessing the effects of fermented dairy products *per se*.

**Table 1:** PICOS criteria for inclusion and exclusion of studies.

Criteria	Inclusion and exclusion criteria for intervention studies	Inclusion and exclusion criteria for observational studies
Population	Individuals aged ≥18 years, generally healthy, without chronic diseases (except untreated hypercholesterolemia), and not using medications known to affect lipid metabolism (e.g., statins, fibrates) were eligible. Studies with pregnant or lactating women were excluded.	Individuals aged ≥18 years, generally healthy (as defined in the intervention study criteria), or participants from population-based studies (e.g., cohorts, surveys).
Intervention/exposure	Studies with a duration of ≥2 weeks, testing conventional versions of yogurt, kefir, fermented milk, or cheese, were eligible. Studies were excluded if they tested enriched versions of fermented dairy, i.e., products fortified with additional bioactive compounds (e.g., plant sterols, fiber) or probiotic strains after the fermentation process, had modified composition, included butter, cream, or buttermilk (due to unclear fermentation status), tested beverages containing >1.25% alcohol, or involved non-nutritional applications of fermented dairy.	Studies evaluating total intake of fermented dairy products or specific categories (e.g., yogurt, kefir, cheese, fermented milk) were eligible. Studies assessing whole dietary patterns were excluded.
Comparator	No intake, lower or less frequent intake of fermented dairy products, or non-fermented dairy products were accepted as controls.	Lower intake levels of fermented dairy products (total or by category) compared to the exposure group.
Outcomes	Total cholesterol, LDL-c, HDL-c, or triglycerides measured in blood, with values reported at both baseline and post-intervention, or reported as changes.	Associations between fermented dairy intake and total cholesterol, LDL-c, HDL-c, or triglycerides levels, or prevalence or incidence of cardiovascular diseases.
Study design	Controlled and non-controlled trials (pre–post intervention assessments) were eligible. Reviews and conference abstracts were excluded.	Observational studies, including cross-sectional and prospective cohort designs, were eligible. Reviews and conference abstracts were excluded.

HDL-c, high-density lipoprotein cholesterol; LDL-c, low-density lipoprotein cholesterol.

#### 2.1.4 Data extraction

Two reviewers independently extracted data using a standardized MS Excel form developed specifically for this review (Supplementary Table 2). Discrepancies were resolved through discussion and consensus or, if necessary, by consultation with a third reviewer. In total, detailed data extraction was completed for studies that met the criteria for outcome evaluation: 14 interventional studies following a PICO format (trials typically randomized and controlled with a comparator), 37 interventional studies following a PIO format (non-controlled trials), and 17 observational studies.

#### 2.1.5 Data synthesis

A qualitative synthesis of the evidence was employed, structured by study design (intervention studies and observational studies) and type of fermented dairy product. For the studies meeting the PICO criteria, results were grouped by product type (e.g., yogurt, kefir, fermented milk, and cheese) and discussed in further detail, considering other relevant study characteristics such as population, comparator, and study duration, when possible. This was followed by a synthesis of studies meeting the PIO criteria, in which patterns were analyzed across studies to identify potential consistencies. Data extracted for primary outcomes (total cholesterol, LDL, HDL, and triglycerides) were summarized based on reported mean changes from baseline or between-group differences, as provided. Secondary outcomes, such as inflammatory markers, glucose and insulin concentrations, blood pressure, and anthropometric parameters, were included where available.

Observational studies were examined for associations between fermented dairy intake and absolute levels (cross-sectional studies) or changes (prospective studies) in blood lipids, as well as associations with CVDs. The outcomes were analyzed in relation to total fermented dairy intake or product categories (e.g., yogurt, cheese). Adjusted hazard ratios (HR), relative risks (RRs), and odds ratios (ORs) were extracted along with the covariates used in multivariable models. For all types of studies included, heterogeneity across studies was addressed qualitatively by comparing populations, intervention characteristics, and reported outcomes.

#### 2.1.6 Risk of bias

Risk of bias was assessed for each controlled trial that fulfilled the PICO criteria, using the Revised Cochrane Risk of Bias tool for Randomized Trials (RoB 2) (24). This tool evaluates five domains: the randomization process, deviations from intended interventions, missing outcome data, measurement of the outcome, and selection of the reported result. Each domain, as well as the overall assessment for each study, was rated as having a low risk of bias, some concerns, or a high risk of bias. The assessment was performed independently by two reviewers. Discrepancies were resolved through discussion and consensus or, if necessary, by consultation with a third reviewer. The figure illustrating the domain-specific risk of bias was generated using the RobVis tool (24).

### 2.2 Non-systematic part of the review

In addition to the formal systematic review of human studies, a narrative (non-systematic) approach was performed. In alignment with EFSA guidance, this narrative synthesis was used to integrate contextual data: (a) the characteristics of the fermented foods studied, (b) supportive evidence on the bioavailability of relevant compounds, (c) plausible mechanisms of action, and (d) the safety of fermented food consumption.

Characteristics of the fermented dairy products were systematically extracted into a harmonized table (Tables 2, 3), which included details on product type, origin, starter cultures, raw materials, manufacturing processes, chemical composition, microbial counts, and analytical methods.

**Table 2:** Characteristics of yogurt from the reviewed studies.

References	Type of product	Source of product	Starter cultures	Raw material	Manufacturing process	Specification/chemical composition of products	Microbiota counts in the final products	Analytical methods
Ataie-Jafari et al., 2009 (74)	Yogurt	Ordinary yogurt: commercial Probiotic yogurt: NR	Ordinary yogurt: *Streptococcus thermophilus* and *Lactobacillus delbrueckii* subsp. *Bulgaricus* probiotic yogurt: *S. thermophilus* and *L. delbrueckii* subsp. *Bulgaricus*, *L. acidophilus* and *Bifidobacterium lactis*	Ordinary yogurt: NR Probiotic yogurt: 2.5% fat milk	NR	NR	Ordinary yogurt: NR Probiotic yogurt: >10^6^ CFU of both *L. acidophilus* and *B. lactis*/g	NR
Anderson and Gilliland, 1999 (71)	Yogurt	All: laboratory	L1 FM: 1.0% (v/v) *L. acidophilus* L1 and 0.1% (v/v) *S. thermophilus* MUH34 ATCC FM: 1.0% (v/v) *L. acidophilus* ATCC 43121 and 0.1% (v/v) *S. thermophilus* MUH34 Placebo: 1.0% (v/v) *S. thermophilus* MUH34	All: demineralized water containing 12% (w/v) skimmed milk powder and 0.4% (w/v) gelatin (Bloom 240)	L1 FM: fermentation (37 °C, 18 h) until pH ~ 4.6, packing, storage (7 °C) ATCC FM: fermentation (37 °C, 18 h) until pH ~ 4.6, packing, storage (7 °C) Placebo: fermentation (37 °C, 24 h) until pH ~ 4.6, packing, storage (7 °C)	NR	Study I: L1 FM: 3.5×10^7^ (day 1)-3.9×10^6^ (day 7) *L. acidophilus* L1 CFU/g ATCC FM: 1.8×107 (day 1)-6.7×106 (day 7) *L. acidophilus* ATCC 43121 CFU/g Placebo: NR Study II: L1 FM: 5.1–7.6×107 (day 1)-4.2–5.9×106 (day 7) *L. acidophilus* L1 CFU/g placebo: NR	MA (CM) for *L. acidophilus*
Rizkalla et al., 2000 (80)	Non-fat yogurt; fresh and heated	All: commercial	*L. bulgaricus* and *S. thermophilus*	NR	All: production according to French regulations	All: E: 193 kJ, C: 6.4 g, F: 0.1 g, P: 4.9 g, and Ca: 300 mg per 100 g	Fresh yogurt: ≥10^7^ *L. bulgaricus* and *S. thermophilus* CFU/g Heated yogurt: ≤10^2^ *L. bulgaricus* and *S. thermophilus* CFU/g	NR
Klein et al., 2008 (97)	Yogurt	Commercial	*L. bulgaricus* and *S. thermophilus*	NR	NR	NR	NR	NR
Olmedilla-Alonso et al., 2017 (73)	Yogurt	Whole cow’s milk yogurt: commercial Whole ewe’s milk yogurt: laboratory Semi-skimmed ewe’s milk yogurt: laboratory	Whole cow’s milk yogurt: NR Whole ewe’s milk yogurt: *S. thermophilus* and *L. bulgaricus* Semi-skimmed ewe’s milk yogurt: *S. thermophilus* and *L. bulgaricus*	Whole cow’s milk yogurt: whole cow’s milk (3.0% milk fat) Whole ewe’s milk yogurt: whole ewe’s milk (5.8% milk fat) Semi-skimmed ewe’s milk yogurt: semi-skimmed ewe’s milk (2.8% milk fat)	Whole cow’s milk yogurt: NR Whole ewe’s milk yogurt: standardisation and pasteurization (80 °C, 30 min) of raw milk, filtration, cooling (42–43 °C), addition of starter cultures, fermentation (42 °C) until pH = 4.6, storage (4 °C) Semi-skimmed ewe’s milk yogurt: standardisation and pasteurization (80 °C, 30 min) of raw milk, filtration, skimming, cooling (42–43 °C), addition of starter cultures, fermentation (42 °C) until pH = 4.6, storage (4 °C)	Whole cow’s milk yogurt: E: 52.9 kcal, F: 3.0 g (SFA: 73.87 g, MUFA: 23.35 g, PUFA: 2.78 g, SCFA: 11.31 g, MCFA: 19.73 g, LCFA: 68.96 g, CLA: 0.26 g per 100 g fat; n3: 0.42 g; n6/n3: 5.00; AI: 6.68), P: 3.2 g, TS: 11.2 g, Ca: 108.13 mg, K: 138.00 mg, Mg: 8.43 mg per 100 g Whole ewe’s milk yogurt: E: 88.5 kcal, F: 5.8 g (SFA: 79.59 g, MUFA: 17.96 g, PUFA: 2.45 g, SCFA: 21.17 g, MCFA: 21.97 g, LCFA: 56.86 g, CLA:0.24 g per 100 g fat; n3: 0.76 g; n6/n3: 2.13; AI: 7.49), P: 5.8 g, TS: 16.7 g, Ca: 201.22 mg, K: 124.30 mg, Mg: 17.88 mg per 100 g Semi-skimmed ewe’s milk yogurt: E: 62.2 kcal, F: 2.8 g (SFA: 77.85 g, MUFA: 19.38 g, PUFA: 2.77 g, SCFA: 20.26 g, MCFA: 20.99 g, LCFA: 58.75 g, CLA: 0.27 g per 100 g fat; n3: 0.87 g; n6/n3: 2.07; AI: 7.37), P: 5.9 g, TS: 14.1 g, Ca: 206.31 mg, K: 126.43 mg, Mg: 17.81 mg per 100 g	NR	MA (CM); lactose measurement; pH measurement; F, P and TS contents determination (ISO methods); Ca, Mg, and K: Ion Chromatography; FA analysis: GC–MS
de Roos et al., 1999 (92)	Non-fat yogurt	Commercial	*S. thermophilus*	NR	NR	NR	NR	MA (CM)
Georgakouli et al., 2016 (79)	Yogurt (Greek, 2% F)	Commercial	*L. bulgaricus* and *S. thermophiles*	Semi-skimmed cow’s milk	Pasteurization of raw milk (85 °C, 5 min), cooling (42 °C), addition of starter culture, fermentation (42 °C, 8–10 h) until pH = 4.3, storage (4 °C, 2 m)	E: 52 kcal, C: 4.6 g, F: 2 g and P: 4.0 g per 100 g	~10^9^ (fresh)-10^8^ (2 m) *S. thermophillus* CFU/g; ~10^6^ (fresh)-10^3^ (2 m) *L. bulgaricus* CFU/g	MA (CM); total titratable acidity measurement
Chang et al., 2011 (75)	Yogurt	Commercial	*S. thermophilus*, *L. acidophilus* and *B. infantis*	Raw milk mixed with skimmed milk powder	Addition of starter culture, addition of lactase, fermentation (36–38 °C, 7–9 h)	E: 90kcal, C: 11.1 g, F: 3.0 g, P: 3.7 g, ash: 0.8 g, Na: 60 mg and K: 160 mg per 150 mL	*S. thermophilus:* ≥3 × 10^9^ CFU/g, *L. acidophilus:* ≥3 × 10^9^ CFU/g and *B. infantis:* ≥1 × 10^10^ CFU/g	NR
Mensink et al., 2002 (100)	Low-fat yogurt (0.7% F)	NR	NR	NR	NR	C: 12.8 g, milk F: 0.2 g and P: 3.7 g and rapeseed oil FA: 0.5 g per 100 g	NR	NR
Seppo et al., 2007 (96)	Low-fat milk products; yogurt and yogurt single-shot drink	NR	NR	NR	NR	Yogurt: E: 260 kJ, C: 12 g, F: 0.1 g and P: 3.0 g per 100 g Yogurt single-shot drink: E: 300 kJ, C: 14 g, F: 0.1 g and P: 3.9 g per 100 g	NR	NR
Nishiyama et al., 2018 (40)	Yogurt	Commercial	*S. thermophilus and L. delbrueckii* subsp. *Bulgaricus* and *L. acidophilus*	NR	NR	NR	NR	NR
Shafie et al., 2022 (94)	Yogurt	Commercial	*L. delbrueckii* subsp. *Bulgaricus* and *S. thermophiles*	NR	NR	NR	NR	NR
Štšepetova et al., 2023 (53)	Yogurt	NR	Plain: *S. thermophilus* and *L. delbrueckii* subsp. *Bulgaricus* Probiotic: *S. thermophilus, L. delbrueckii* subsp. *Bulgaricus* and *L. plantarum* Inducia	All: cow’s milk	All: pasteurization of raw milk, addition of starter culture, fermentation, sweetening (5% sugar), packing, storage (2–6 °C)	All: E: 75 kcal, C: 10.2 g, F: 2.2 g (SFA: 1.4 g), P: 3.4 g, L: 26 g and DF: 0.5 g per 100 g	Plain: *S. thermophilus:* 4.5 × 10^7^ CFU/mL and *L. delbrueckii* subsp. *Bulgaricus:* 4.4 × 10^8^ CFU/mL Probiotic: *S. thermophilus:* 4.5 × 10^7^ CFU/mL*, L. delbrueckii* subsp. *Bulgaricus:* 4.4 × 10^8^ CFU/mL and *L. plantarum* Inducia: ~2.6 × 10^7^ CFU/mL	NR
Watanabe et al., 2018 (99)	Yogurt	NR	NR	NR	NR	E: 72 kcal, C: 10.2 g, F: 0 g and P: 6.8 g per 150 g	NR	NR
Detopoulou et al., 2021 (81)	Low-fat strained yogurt	Commercial	NR	NR	NR	E: 85kcal per 150 g; C: 14 g (S: 13 g), F: 1 g (SFA: 0.6 g), P: 5 g and Na: 0.1 g per 100 g; 16% (w/w) strawberry supplement; phenolic compounds: 2.1 mg/ g lyophilized sample	NR	Physicochemically tested for the concentration in macronutrients and microbial parameters by the Greek dairy industry
Songisepp et al., 2022 (76)	Yogurt	NR	Plain: *S. thermophilus* and *L. delbrueckii* subsp. *Bulgaricus* Probiotic: *S. thermophilus, L. delbrueckii* subsp. *Bulgaricus* and *L. plantarum* Inducia	All: cow’s milk	All: pasteurization of raw milk, cooling (35–43 °C), addition of starter culture, fermentation until pH = 4.2–4.5, cooling (23–27 °C), sweetening (5% sugar), packing, storage (2–6 °C)	All: E: 75 kcal, C: 10.2 g, F: 2.2 g (SFA: 1.4 g), P: 3.4 g, L: 26 g and DF: 0.5 g per 100 g	NR	NR
Agerholm-Larsen et al., 2000 (68)	Yogurt	All: commercial	G: 1 strain of *Enterococcus faecium* (human species) and 2 strains of *S. thermophilus* (Gaio) StLa: 2 strains of *S. thermophilus* and 2 strains of *L. acidophilus* StLr: 2 strains of *S. thermophilus* and 1 strain of *L. rhamnosus*	NR	NR	E: 54 kcal, C: 3.3 g, F: 1.7 g (1% milk F, 0.7% rapeseed oil), P: 5.4 g and Ch: 3–4 mg per 100 g	G: *E. faecium*: 6×10^7^ CFU/mL and *S. thermophilus*: 1×10^9^ CFU/mL StLa: *S. thermophilus*: 10×10^7^ CFU/mL and *L. acidophilus*: 2×10^7^ CFU/mL StLr: *S. thermophilus*: 8×10^8^ CFU/mL and *L. rhamnosus*: 2×10^8^ CFU/mL	NR
Nestel et al., 2013 (26)	Fermented dairy diet: cheddar cheese and full-cream yogurt; low-fat dairy diet: <1% F yogurt	NR	NR	NR	NR	NR	NR	NR
Hepner et al., 1979 (33)	Yogurt; unpasteurized and pasteurized	All: commercial	All: *L. bulgaricus* and *S. thermophilus*	All: skim milk	NR	NR	NR	NR
Sadrzadeh-Yeganeh et al., 2010 (35)	Yogurt (2.5% F)	NR	*L. bulgaricus* and *S. thermophilus*	NR	NR	NR	10^6^–10^7^ CFU of *S. thermophilus* and *L. bulgaricus*	NR
Antonopoulou et al., 2022 (34)	Low-fat strained yogurt	Commercial	NR	NR	Pasteurization of raw milk, cooling, addition of starter culture, fermentation	E: 85kcal per 150 g; C: 14 g (S: 13 g), F: 1 g (SFA: 0.6 g), P: 5 g and Na: 0.1 g per 100 g; flavored with strawberry	NR	NR
Dawczynski et al., 2013 (98)	Yogurt (3.5% w/w F)	Commercial	NR	NR	NR	NR	NR	NR
Volpe et al., 2001 (82)	Low-fat low-lactose yogurt	NR	NR	NR	NR	E: 384 kJ, C: 15 g, F: 2 g, P: 3 g and Ch: 5 mg per 100 mL	NR	NR
Jones et al., 2012 (84)	Yogurt	Commercial	NR	NR	NR	E: C: 11.5 g, L: 1.3 g and P: 7.9 g per 125 g	1.25×10^9^ CFU yogurt bacteria/125 g	NR
Hyun et al., 2005 (95)	Low-fat yogurt	Commercial	NR	NR	NR	E: 276 kJ, C: 13 g, F: 0.07 g and P: 3.4 g per 100 mL	NR	NR
Sialvera et al., 2012 (39)	Yogurt mini drink	NR	NR	NR	NR	NR	NR	NR
Vásquez-Trespalacios and Romero-Palacio, 2014 (93)	Yogurt drink	Commercial	NR	NR	NR	E: 54 kcal, C: 7.3 g, F: 1.5 g and P: 2.8 g per 100 mL	NR	NR
Niittynen et al., 2008 (83)	Low-fat yogurt drink	NR	NR	NR	NR	E: 384 kJ, C: 15 g, F: 2 g, P: 3 g and Ch: 5 mg per 100 g	NR	NR
Oosthuizen et al., 1998 (38)	Frozen yogurt	NR	NR	skim milk	NR	E: 948 kJ, palmitic acid: 0.84 g, stearic acid: 0.30 g, oleic acid: 0.70 g, linoleic acid: 0.04 g and *α*-linoleic acid: 0.04 g per 175 g	NR	NR
Lee et al., 2017 (72)	Yogurt smoothie	all: laboratory	YS: *S. thermophilus* and *L. delbrueckii* subsp*. Bulgaricus* PRE: *S. thermophilus, L. delbrueckii* subsp*. Bulgaricus* and *B. animalis* subsp*. lactis* BB-12	NR	NR	All: E: 220 kcal, C: 45 g (S: 31 g, DF: 1 g), F: 2.5 g SFA: 1.5 g, P: 7 g, Na: 90 mg and Ch: 10 mg per 8 oz	YS: NR PRE: 3.6 CFU *B. animalis* subsp*. lactis* BB-12/ 8 oz	NR

NR, Not reported; E, energy; C, carbonhydrates; F, fat; P, protein; TS, total solids; L, lipids; DF, dietary fiber; S, sugars; Ch, cholesterol; vit., vitamin; FA, fatty acids; SFA, saturated fatty acids; MUFA, monounsaturated fatty acids; PUFA, polyunsaturated fatty acids; SCFA, short-chain fatty acids; MCFA, medium-chain fatty acids; LCFA, long-chain fatty acids; CLA, conjugated linoleic acid; AI, atherogenic index = [C12:0 + (C14:0 × 4) + C16:0]/(Total unsaturated fatty acids); NDF, neutral detergent fibre; NSC, non-structural carbohydrates; PC, total peptide concentration; VPP, tripeptide Val-Pro-Pro; IPP, tripeptide Ile-Pro-Pro; MA (CM), microbiologiacal analysis (classical microbial counts methods).

**Table 3:** Characteristics of kefir, cheese and other fermented dairy products from the reviewed studies.

References	Type of product	Source of product	Starter cultures	Raw material	Manufacturing process	Specification/chemical composition of products	Microbiota counts in the final products	Analytical methods
Bourrie et al., 2023 (42)	Kefir (1% F)	1 laboratory (pitched kefir) and 1 commercial	Pitched: *Acetobacter pasteurianus, Lactococcus lactis, Leuconostoc mesenteroides, Lentilactobacillus kefiri, L. kefiranofaciens, Pichia fermentans, Saccharomyces cerevisiae, Kazachstania unispora,* and *Kluyveromyces marxianus* (previously isolated from a traditional kefir grain) *Commercial: L. lactis, Lacticaseibacillus rhamnosus, S. diacetylactis, Lactiplantibacillus plantarum, Lacticaseibacillus casei, Saccharomyces florentinus, Leuconostoc mesenteroides* subsp*. cremoris, B. longum, B. breve, L. acidophilus, B. lactis,* and *L. reuteri*	Pitched: 2% fat milk Commercial: NR	Pitched: addition of starter culture, fermentation (22 °C, 18 h), storage (4 °C) Commercial: NR	NR	Pitched kefir: NR Commercial: 8.0×10^6^ CFU/mL	NR
Yilmaz and Arslan, 2022 (41)	Kefir	NR	NR	NR	NR	NR	Lactobacillus: 10.54 log (CFU)/mL, Lactococcus: 10.62 log (CFU)/mL, Total yeast: 2.69 log (CFU)/mL, *L. acidophilus*: 8.25 log (CFU)/mL, B.: 7.78 log (CFU)/mL *L. kefiri, L. kefiranofaciens* subsp*. kefiranofaciens, L. kefiranofaciens* subsp*. Kefirgranum, L. parakefiri, L. acidophilus, L. casei, L. reuteri, L. bulgaricus, L. helveticus, L. fermentum, Leuconostoc mesentereoides, Lactococcus lactis, S. thermophilus, B. bifidum, A. pasteurianus, K. marxianus, S. scerevisiae, K. slactis*	MA (CM) and qReal-Time PCR (Roche)
Fathi et al., 2017 (30)	Kefir	Commercial	NR	NR	NR	E: 118 kcal, C: 10 g, F: 5 g, P: 8 g, and Ca: 300 mg per serving (250 cc)	NR	NR
Bellikci-Koyu et al., 2022 (25)	Kefir	NR	*Lactococcus lactis* subsp*. lactis, Lactococcus lactis* subsp*. cremoris, L. lactis* subsp*. diacetylactis, Leuconostoc mesenteroides* subsp*. cremoris, L. kefiri, K. marxianus, S. unisporus*	NR	Pasteurization of raw milk (80–85 °C, 10 min), cooling (25 °C), addition of starter culture (3.25%), fermentation (18 h) until pH = 4.7, packing, storage (4 °C)	NR	Lactobacillus spp., Leuconostoc spp., and Lactococcus spp.: minimum 10^6^ CFU/g; Yeasts: 10^5^ CFU/g *Lactococcus lactis* subsp*. lactis, Lactococcus lactis* subsp*. cremoris, Lactococcus lactis* subsp*. diacetylactis, Leuconostoc mesenteroides* subsp*. cremoris, L. kefyr, K. marxianus,* and *S. unisporus*	NR
Pražnikar et al., 2020 (29)	Kefir	Commercial	NR	Full-fat cow milk (3.5% fat)	Pasteurization of raw milk, addition of starter culture (10% wt/v), fermentation (24 °C, 24 h), filtration	E: 239 kcal, C: 3.9 g (S: 3.9 g), F: 3.2 g (SFA: 2.4 g), P: 3.2 g, Na: 0.1 g and Ca: 0.12 g per 100 g	Bacterial isolates (~90%): *Lactobacillus* (*L. parakefiri, L. kefiri,* and *L. kefiranofaciens* ssp*. kefirgranum*); bacterial isolates (~10%): cocci; Yeasts (predominant species): *K. marxianus, Kazachstania exigua,* and *Rhodosporidium kratochvilovae*	NR
Bellikci-Koyu et al., 2019 (36)	Kefir	Laboratory	*Lactococcus lactis* subsp*. lactis, Lactococcus lactis* subsp*. cremoris, Lactococcus lactis* subsp*. diacetylactis, Leuconostoc mesenteroides* subsp*. cremoris, L. kefir, K. marxianus,* and *S. unisporus*	3.5% full-fat milk	Homogenization and pasteurization (85 °C) of raw material, addition of starter culture, fermentation, storage (4 °C, 1–4 d)	NR	NR	NR
St-Onge et al., 2002 (31)	Kefir	Commercial	NR	NR	NR	E: 287 kcal, C: 31.2 g, F: 7.6 g, P: 23.6 g, and Ch: 31 mg per 500 mL	Bacterial: 10^9^ CFU	NR
Santurino et al., 2020 (49)	Goat cheese	Commercial	NR	Goat’s milk	According to the process of the Protected Denomination Origin (PDO) ‘Queso de Murcia’ regulatory board; pasteurization of milk, ripening (21 d, 10 °C), vacuum package (shelf-life~6 months)	E: 379.8 kcal, C: <0.5 g, F: 33.28 g, P: 18.6 g, TS: 56.82, P: 400 mg, Ca: 410 mg, Mg: 30 mg, Na: 630 and vit. D: 0 < 1 μg per 100 g; fatty acid profile	NR	F extraction; FA methyl esters analysis (ISO method); Triacylglycerides and Ch determination; Lipid classes composition by HPLC-Evaporative Light Scattering Detection (ELSD)
Feeney et al., 2018 (46)	Diet with full-fat Irish white cheddar cheeses (120 g)	NR	NR	NR	Ripening (8–12 m)	NR	NR	NR
Nilsen et al., 2015 (44)	Chesses; Gamalost and Norvegia	All: commercial	NR	NR	Gamalost: ripening for 10 d Norvegia: ripening for ~90 d	Gamalost: E: 213 kcal, C: 1 g, F: 1 g (SFA: 0 g), P: 50 g, Ca: 160 mg, Na: 24 mg, Mg: 13 mg and K: 98 mg per 100 g Norvegia: E: 351 kcal, C: 0 g, F: 27 g (SFA: 17 g), P: 27 g, Ca: 800 mg, Na: 402 mg, Mg: 33 mg and K: 77 mg per 100 g	NR	NR
Raziani et al., 2016 (47)	Cheese; regular-fat: Danbo (25% F) and cheddar (32% F) (REG) and reduced-fat Danbo (13% F) and cheddar (16% F) (RED)	All: commercial	NR	NR	NR	REG: Danbo: E: 528 kJ, C: 0.2 g, F: 10.2 g (SFA: 6.3 g, MUFA: 2.8 g, PUFA: 0.3 g), P: 8.8 g, Ca: 267 mg and Na: 0.6 g per 40 g Cheddar: E: 666 kJ, F: 13.2 g (SFA: 8.1 g, MUFA: 4.6 g, PUFA: 0.7 g), P: 10.0 g, Ca: 285 mg and Na: 0.7 g per 40 g RED: Danbo: E: 378 kJ, C: 0.2 g, F: 5.4 g (SFA: 3.3 g, MUFA: 1.3 g, PUFA: 0.1 g), P: 10.4 g, Ca: 309 mg and Na: 0.6 g per 40 g Cheddar: E: 614 kJ, F: 9.6 g (SFA: 5.6 g, MUFA: 3.3 g, PUFA: 0.3 g), P: 15.2 g, Ca: 285 mg and Na: 0.7 g per 40 g	NR	NR
quiainen et al., 2006 (48)	Cheese	NR	NR	NR	NR	E: 600 kJ, C: 0 g, F: 8.5 g (SFA: 4.5 g) and P: 17 g per 50 g	NR	NR
Raziani et al., 2018 (50)	Cheese; regular-fat: Danbo (25% F) and cheddar (32% F) (REG) and reduced-fat Danbo (13% F) and cheddar (16% F) (RED)	All: commercial	NR	NR	NR	REG: Danbo: E: 528 kJ, C: 0.2 g, F: 10.2 g (SFA: 6.3 g, MUFA: 2.8 g, PUFA: 0.3 g), P: 8.8 g, Ca: 267 mg and Na: 0.6 g per 40 g Cheddar: E: 666 kJ, F: 13.2 g (SFA: 8.1 g, MUFA: 4.6 g, PUFA: 0.7 g), P: 10.0 g, Ca: 285 mg and Na: 0.7 g per 40 g RED: Danbo: E: 378 kJ, C: 0.2 g, F: 5.4 g (SFA: 3.3 g, MUFA: 1.3 g, PUFA: 0.1 g), P: 10.4 g, Ca: 309 mg and Na: 0.6 g per 40 g Cheddar: E: 614 kJ, F: 9.6 g (SFA: 5.6 g, MUFA: 3.3 g, PUFA: 0.3 g), P: 15.2 g, Ca: 285 mg and Na: 0.7 g per 40 g	NR	NR
Pintus et al., 2013 (51)	Cheese (~ 26% F)	Commercial	None	Ewe’s milk	Heat treatment of milk (35 °C), coagulation (animal rennet), vacuum package, storage (−20 °C)	F: 2.9%, P: 17.9%, TS: 90%, ash: 7.8%. NDF: 24.3% and NSC: 32.7% per dry matter (90%); fatty acid profile	NR	F extraction (ISO method); FA composition analysis (GC); CLA isomers analysis: HPLC-DAD; TG analysis (ISO method); Ch determination: GC-FID
Davis et al., 1993 (52)	Mozzarella cheese	NR	NR	Partial skim milk	Storage (−20 °C)	E: 90 cal (60% cal as F), F: 6 g and P: 9 g per ounce (28 g); Ch: 0.6 mg per g; fatty acid composition	NR	NR
Thorning et al., 2015 (45)	Diet with high-fat cheese	NR	NR	NR	NR	NR	NR	NR
Soerensen et al., 2014 (28)	Diet with semihard cow cheese	NR	NR	Cow’s milk	NR	NR	NR	NR
Dönmez et al., 2014 (43)	Koumiss	Homemade	NR	NR	NR	NR	NR	NR
Richelsen et al., 1996 (32)	Fermented milk product (Gaio)	Commercial	1 strain of *E. faecium* (human species) and 2 strains of *S. thermophilus*	NR	NR	E: 240 kJ, C: 5.4 g, F: 1.5 g (2/3 milk F, 1/3 soybean F), P: 4.9 g, Ch: 5 mg, vit. E: 0.5 mg and vit. C: 10 mg per 100 g	10^5^–10^9^ CFU/mL: *E. faecium* and 5–20×10^8^ CFU/mL *S. termophilus*	NR
Agerbaek et al.,1995 (68)	Fermented milk product (Gaio)	Commercial	1 strain of *E. faecium* (human species) and 2 strains of *S. thermophilus*	NR	NR	E: 230 kJ, C: 6.0 g, F: 1.3 g and P: 4.5 g per 100 g	~2×10^8^ CFU/mL: *E. faecium* and ~7×10^8^ CFU/mL *S. termophilus*	NR
Usinger et al., 2010 (27)	Fermented milk product (Cardio04)	NR	*L. helveticus*	Reconstituted 9% (w/t) skim milk	Pasteurization of milk (90 °C, 60 min), cooling (43 °C), addition of starter culture (1% w/t), fermentation (37 °C, 7 h), addition of glucose and natural mango fruit flavour, pasteurization, homogenization	PC: 4.7 mg per mL; VPP: 2.5 mg and IPP: 1.1 mg per 300 mL	NR	NR

NR, Not reported; E, energy; C, carbonhydrates; F, fat; P, protein; TS, total solids; L, lipids; DF, dietary fiber; S, sugars; Ch, cholesterol; vit., vitamin; FA, fatty acids; SFA, saturated fatty acids; MUFA, monounsaturated fatty acids; PUFA, polyunsaturated fatty acids; SCFA, short-chain fatty acids; MCFA, medium-chain fatty acids; LCFA, long-chain fatty acids; CLA, conjugated linoleic acid; AI, atherogenic index = [C12:0 + (C14:0 × 4) + C16:0]/(Total unsaturated fatty acids); NDF, neutral detergent fibre; NSC, non-structural carbohydrates; PC, total peptide concentration; VPP, tripeptide Val-Pro-Pro; IPP, tripeptide Ile-Pro-Pro; MA (CM), microbiologiacal analysis (classical microbial counts methods).

## 3 Results and discussion

### 3.1 Identification of pertinent human efficacy studies

#### 3.1.1 Intervention studies

**Figure 1:**
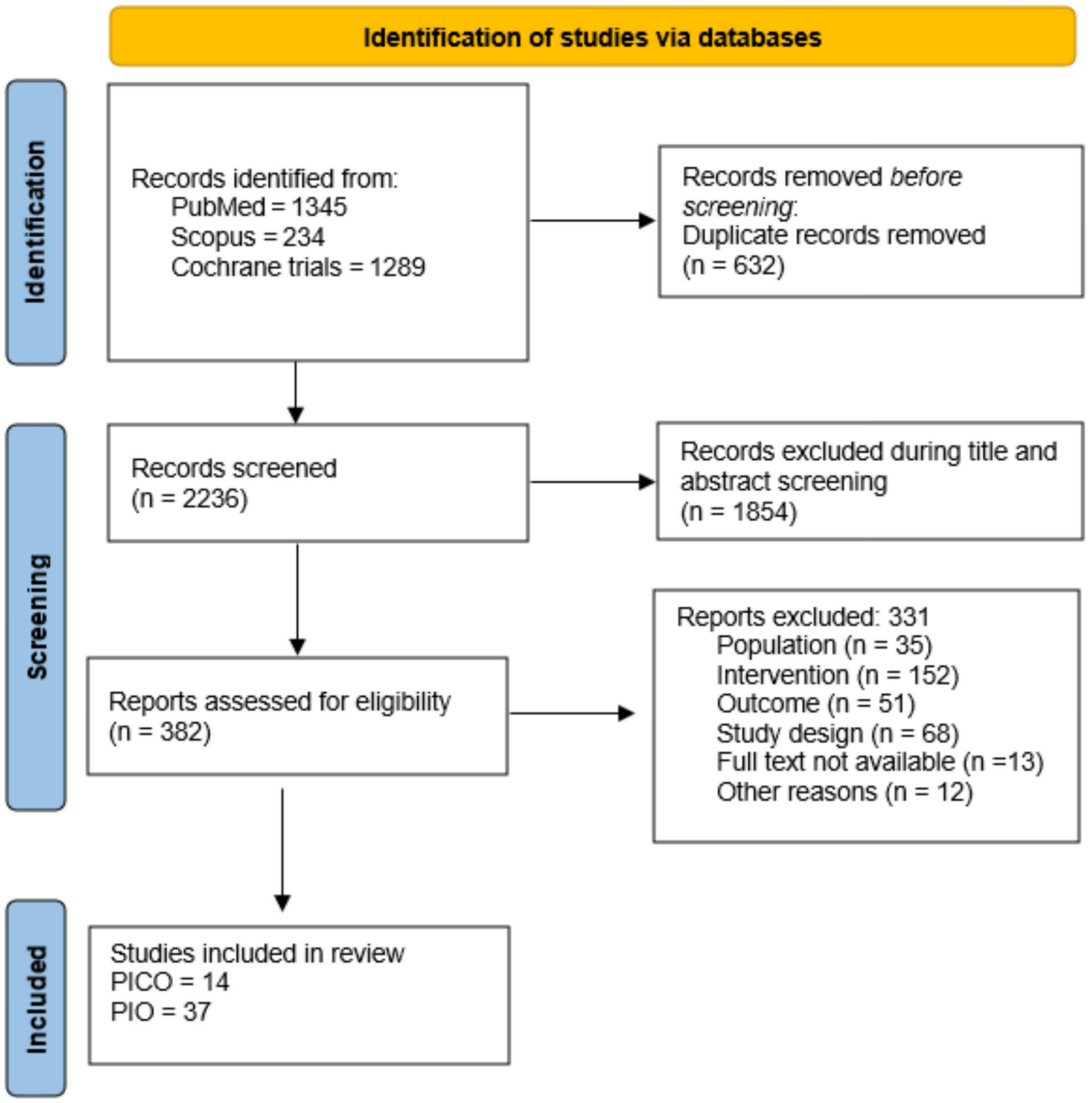
PRISMA flow diagram for the systematic review.

The search strategy retrieved 2,868 articles from databases (Figure 1), with no additional articles identified through other sources. After removing duplicates (*n* = 632), 2,236 articles were screened by title and abstract, with 382 articles selected for full-text assessment. During full-text review, 331 articles were excluded for various reasons, the most frequent being unsuitable intervention (*n* = 152), study design (*n* = 68), outcome (*n* = 51), and ineligible populations (*n* = 35). The final qualitative synthesis included 14 studies meeting PICO criteria, 37 studies meeting PIO criteria, and 17 observational studies.

Given the greater relevance of the PICO studies to the objective of this review, we begin by presenting their characteristics (Table 4). These studies were published between 1979 and 2022 and conducted across a range of countries. Denmark contributed the largest number of studies (*n* = 5), followed by Iran (*n* = 2) and Turkey (*n* = 2), while the USA, Greece, Canada, Slovenia, and Australia each contributed one. The most commonly studied dairy products were kefir (*n* = 5) (24–28), yogurt (*n* = 4) (26); and fermented milk (*n* = 4) (29–32). Cheese was only assessed in one study (28). Collectively, the included studies assessed the primary outcomes of our review – the cardiometabolic markers TG, total cholesterol, HDL and/or LDL cholesterol. Some also examined other relevant variables, including fasting blood glucose, insulin, CRP (or hs-CRP), IL-6, TNFα, blood pressure, and BMI, which were considered secondary outcomes.

**Table 4:** Characteristics of the PICO studies included in the review.

References	Targeted population	Group	N. of Ind.; F/M	Age	BMI	Country	Study design	Intervention duration	Diet	Product	Dose	Strain	Compliance
Agerbaek et al., 1995 (68)	Danish non-obese, normocholesterolemic males	Gaio	0/29	44	24.3 ± 2.0	DEN	Parallel RCT (double blind)	6 weeks	Not to change their ordinary diet, exercise, tobacco/alcohol consumption	fermented milk	200 mL daily	*Enterococcus faecium* and two strains of *Streptococcus thermophilus*	100%
		Placebo	0/28		24.1 ± 1.7							Chemically acidified milk (glucolactone)	
Richelsen et al., 1996 (32)	HI aged 50-70yo, BMI < 32 kg/m^2^, no medication affecting plasma lipids	I	44; 20/24	M: 59.5 ± 1.22/F: 59.7 ± 1.4	M: 25.29 ± 0.44/F: 24.7 ± 0.75	DEN	pRCT	6 months	NR	Fermented milk	200 mL daily	*Enterococcus faecium* and two strains of *Streptococcus thermophilus*	87/90 Ind completed the study (41F/46M)
		C	43; 21/22	M: 58.5 ± 1.23/F: 59.8 ± 1.12	M: 25.6 ± 0.6/F: 25.1 ± 0.7					Placebo milk	200 mL daily	No bacterial cultures	
Hepner et al., 1979 (33)	HI, no history of CVD or cerebrovascular, or gallbladder disease, no medication, but 12 of the subjects were tobacco smokers	I	31; 16/15	26 ± 3; 35 ± 10; 39 ± 12	NR	USA	pRCT	12 weeks (3 × 4 weeks) Study I: 4-week intervention followed by 4-week washout period and another 4-week intervention/study II: all groups 12 weeks except for group *F* = 6 weeks	NR	Study I (Group A: unpasteurized yogurt)/Study II (Group C&D: unpasteurized yogurt& pasteurized yogurt)	NR	NR	Only 3 subjects had serum TG > 200 mg/l00 (2 in group C and group D) and 9 subjects in study II with serum TC > 280 mg/100 ml
		C	18; 10/8	27 ± 4; 37 ± 12	NR					Butterfat milk (group B for study I and group E for study II)	NR	NR	NR
Sadrzadeh-Yeganeh et al., 2010 (35)	Women, 19 to 49 yo, TC < 6.2 mmol/L, TAG levels < 2.3 mmol/L and a BMI < 30 kg/m^2^	I	30F	32	23 ± 2.4	IRN	pRCT	6 weeks	No alteration of exercise routine or regular diet, no consumption of any yogurt other than the one provided. Refrain from consuming any other probiotic and fermented products	Conventional yogurt	300 g daily	*Lactobacillus bulgaricus* and *Streptococcus thermophilus*	Compliance monitored once per week through phone interviews. One C subject removed because of ATB
		C	30F	34.7	28.3 ± 3					No consumption	NA	NA	
Antonopoulou et al., 2022 (34)	Healthy adults aged 35–65 years old	I	28; 14/14	48.8 ± 8.9	28.6 ± 3.8	GRC	pRCT	8 weeks	No changes of regular diet during the study period. Change in dietary intake assess by FQ and 24 h recalls	Plain yogurt	150 g daily	NR	Assessed biweekly; two participants from intervention group were considered as dropouts as they did not complete the intervention.
		C	30; 14/16	49.4 ± 8.8	27.7 ± 6.4					No consumption (consume at most one yogurt every two weeks)	NA	NA	
Soerensen et al., 2014 (28)	HI, 18–50 years old, BMI of 20–28 kg/m^2^, stable weight	I	15 M	27.7 ± 4.8	23.1 ± 2.3	DEN	Crossover RCT	14 days	NR	Milk	670 mL daily	NR	Measure of the excreted calcium in urine and feces
		I								Cheese	120 g daily	NR	
		C								Non dairy	NA	NA	
Bellekçi-Koyu et al., 2019 (36)	18–65 years old with metabolic syndrome. Eligibility confirmed with a physical examination and nutritional assessment	I	12; 10/2	52.00 (47.50–60.50)	30.67 (26.94–34.66)	TUR	pRCT	12 weeks	Regular diet. Additional products that contain probiotics were not allowed during the intervention period	Kefir	180 mL daily	NR	Consumption of >80% of the test drinks. 22/40 participants completed the study.
		C	10; 6/4	53.00 (45.00–60.00)	32.38 (29.18–34.59)					Milk	180 mL daily	NA	
St-Onge et al., 2002 (31)	13 healthy mildly hypercholesterolemic male subjects, included total serum cholesterol levels 6–10 mmol/L, non-smoking, BMI from 26 to 38 kg/m^2^ (30.2 ± 4.4 kg/m^2^). Mean serum cholesterol levels at screening were 6.54 ± 0.78 mmol/L	I	13; NA/13	47 ± 9 (27–61)	30.2 ± 4.4	CAN	Crossover, randomized placebo-controlled study	4 weeks	regular diet	Kefir	500 mL daily breakfast	NR	NR
		C								Milk	500 mL daily breakfast	NA	
Usinger L. et al, 2010 (27)	Individuals with prehypertension or borderline hypertension without cardiac or renal disease, diabetes, antihypertensive treatment, pregnancy or milk allergy	I	15; NR/NR	Study I: 54 ± 12; Study II: 52 ± 10	Study I: 27 ± 4; Study II: 26 ± 4	DEN	Prospective Randomized double-blind, placebo-controlled trial	8 weeks	regular diet	Fermented milk, containing VPP (2.5 mg) and IPP (1.1 mg). Tripeptides Val-Pro-Pro (VPP) and Ile-Pro-Pro (IPP)	Study I: 300 mL daily Study II: 150 mL daily	*Lactobacillus helveticus*	Study I: 3 were excluded: 1 had abnormal blood samples, 1 had atrial fibrillation, and 1 incomplete data (this is for 300 ml) Study II: 2 were excluded: 1 had grade II hypertension, 1 withdrawal
		C	15; NR/NR	Study I: 54 ± 11; Study II: 54 ± 11	Study I: 26 ± 4; Study II: 26 ± 4					Artificially Acidified Milk	Study I: 300 mL daily; Study II: 150 mL daily	NA	
Jenko Praznikar Z, et al., 2020 (29)	Overweight adults with no acute or chronic diseases, no gastrointestinal diseases or endocrine disorders, no drug use for lipid disorders or anti-inflammatory drugs, no nutritional supplements, antibiotics, not pregnant, and not lactating	I	28; 15/13	45.8 ± 8.4	29.1 ± 4.6	SVN	Crossover intervention study	21 days	regular diet	Kefir	300 mL daily	*Lactobacillus parakefiri*, *Lactobacillus kefiri*, *Lactobacillus kefiranofaciens* ssp. *kefirgranum*, cocci. *Kluyveromyces marxianus*, *Kazachstania exigua*, and Rhodosporidium *kratochvilovae*	one participant dropped out because of chickenpox
		C								Milk	300 mL daily	NA	
Fathi Y, et al., 2017 (30)	Overweight or obese premenopausal women recruited from outpatients referred to the Cardiovascular Research Center (CRC), Shiraz University of Medical Sciences.	I	25; 25/NA	35.2	29.5 (28.5–30.6)	IRN	Single-center, multi-arm, parallel-group, outpatient, randomized controlled trial (RCT)	8 weeks	Study completers had high adherence to study diets. Dietary calcium intake was significantly lower in the control group compared to kefir and milk groups. No significant differences among groups in terms of fiber, energy content, or macronutrient distribution	Kefir	1,000 mL/day	NR	Compliance was defined as intake of ≥90% attributed products. Full compliance of study participants to their allocated intervention was confirmed.
		C	25; 25/NA	37	28.9 (27.9–29.8)					Low fat dairy	1,000 mL/day	NA	
Agerholm-Larsen L, et al., 1999 (37)	Overweight and obese adults (BMI between 25.0 and 37.5 kg/m^2^), aged 18–55 years, including 20 men and 50 women (total 70). “Healthy” and weight-stable (no major health issues, normal blood pressure, etc.).	I	16; 12/4	37.8 ± 2.0	88.9 ± 4.1	DEN	Randomized double-blind, placebo-and compliance-controlled, parallel study	8 weeks	Participants were instructed to maintain their habitual diet and body weight during the intervention. A 7-day weighed dietary record was completed at baseline (week 0) and at week 8.	Fermented milk product GAIO: The intervention group (G) received 450 mL of a fermented milk product containing the CAUSIDO culture daily.	450 mL daily	One strain of *Enterococcus faecium* and two strains of *Streptococcus thermophilus*	Compliance measured at home once every second week during the intervention (weeks 2, 4, 6 and 8) using a 13C-acetate-enriched yogurt and provided pre-and post-consumption breath samples. Compliance ranged 78.6–100%.
		C	10; 7/3	38.3 ± 3.2	85.5 ± 3.7					The comparison group (PP): Two placebo tablets daily, each containing 500 mg of calcium lactate (≈65 mg of calcium). No fermented milk product was consumed in this group.	Two placebo tablets daily, each containing 500 mg of calcium lactate	NA	
Nestel et al., 2013 (26)	Overweight/obese adults	Full-fat fermented dairy	12	60.5 ± 10.7	30.1 ± 2.2	AUS	Randomized crossover trial	12 weeks: 2-week run-in 3 weeks full-fat diet 2 weeks low-fat diet 3 weeks other full-fat diet 2 weeks low-fat diet	Three dietary arms: low fat, Full-fat fermented dairy Full-fat non-fermented dairy (butter, cream, ice cream; 3 weeks) Participants maintained usual caloric intake, adjusting other foods as necessary to keep body weight stable. Macronutrient content (from food diaries): Low-fat diet: ~18% of energy from fat, higher total carbohydrates Fermented diet: ~32% of energy from fat Non-fermented diet: ~35% of energy from fat Sodium and protein levels were modestly different across arms, but all diets were within typical ranges.	Full-fat fermented dairy (cheese + yogurt; 3 weeks)	cheddar cheese (85 g/d) and full-cream yogurt (600 g/d).	NR	The study foods (fermented or non-fermented dairy products) were pre-packaged and delivered fresh twice weekly. Participants were instructed to replace part of their usual diet with these specific dairy items. Unused portions were returned to the investigators to verify that participants were consuming the correct amounts. The paper does not quantify compliance numerically; however, no concerns about compliance were raised, and no major issues were reported.
		Full-fat non-fermented dairy								Full-fat non-fermented dairy (butter, cream, ice cream; 3 weeks). Diet similar for total daily fat amount but no fermented foods (e.g., no yogurt/cheese).	butter (30 g/d), cream (70 mL/d), and small amounts of ice cream.	NR	
		C	10; 7/3	38.3 ± 3.2	85.5 ± 3.7					Low-fat dairy (milk + yogurt; 2 weeks, repeated twice)	1,000 mL daily	NA	
Bellikci-Koyu et al., 2022 (25)	Participants with metabolic syndrome	I	31; 22/9	49.1 ± 8.5 years	32.3 ± 5.8	TUR	Single-center, parallel-group, randomized, controlled trial	12 weeks	regular dietary no restrictions	Kefir	180 mL daily	NR	Compliance was defined as the consumption of >80% of the test drinks during the study period
		C	31; 22/9	50.5 ± 7.5 years	32.9 ± 5.4					Milk	180 mL daily	NA	

	TG		TC		HDL-C		LDL-C		Fasting Blood Glucose		Insulin		CRP	
References	BL	EL	BL	EL	BL	EL	BL	EL	BL	EL	BL	EL	BL	EL
Agerbaek et al., 1995 (68)	1.28 ± 0.37	1.35 ± 0.51	6.08 ± 0.42	5.71 ± 0.49	1.21 ± 0.26	1.23 ± 0.29	4.30 ± 0.34	3.87 ± 0.48	NR	NR	NR	NR	NR	NR
	1.22 ± 0.33	1.14 ± 0.36	5.88 ± 0.47	5.86 ± 0.53	1.32 ± 0.31	1.31 ± 0.26	4.01 ± 0.51	4.03 ± 0.58						
Richelsen et al., 1996 (32)	1.34 ± 0.14	1.21 ± NR	M: 5.37 ± 0.19/F: 6.10 ± 0.22	NR	M: 1.41 ± 0.08/F: 1.84 ± 0.08	NR	M: 3.36 ± 0.16/F: 3.71 ± 0.21	M: 3.06 ± 0.13 (6 months) *F* = 3.4 (1 month from graph)	NR	NR	NR	NR	NR	NR
	1.51 ± 0.18	1.42 ± 0.12	M: 5.53 ± 0.22/F: 6.19 ± 0.27	NR	M: 1.32 ± 0.07/F: 1.95 ± 0.1	NR	M: 3.53 ± 0.21/F: 3.60 ± 0.27	M = 3.54 ± 0.17 (1 month) F–NR	NR	NR	NR	NR	NR	NR
Hepner et al., 1979 (33)	NR	NR	NR	NR	NR	NR	NR	NR	NR	NR	NR	NR	NR	NR
	group B: 0.70 ± 0.09 (MILK)/group B: 0.73 ± 0.07 (yogurt)/ group E: 0.68 ± 0.08	group B: 0.86 ± 0.12 (MILK)/ group B: 0.80 ± 0.14 (yogurt)/group E: 0.77 ± 0.12	group E: 6.15 ± 0.52	group E: 5.79 ± 0.47	NR	NR	NR	NR	NR	NR	NR	NR	NR	NR
Sadrzadeh-Yeganeh et al., 2010 (35)	0.89 ± 0.25	0.92 ± 0.24	4.50 ± 0.66	4.38 ± 0.58	1.31 ± 0.23	1.38 ± 0.23	2.62 ± 0.5	2.59 ± 0.5	NR	NR	NR	NR	NR	NR
	1.08 ± 0.44	1.16 ± 0.38	4.78 ± 0.72	4.96 ± 0.94	1.27 ± 0.31	1.26 ± 0.32	2.85 ± 0.6	2.93 ± 0.63	NR	NR	NR	NR	NR	NR
Antonopoulou et al., 2022 (34)	1.23 (0.93–1.59)	1.12 (0.86–1.56)	5.46 ± 0.80	5.33 ± 0.90	1.40 ± 0.31	1.34 ± 0.36	3.44 ± 0.67	3.39 ± 0.72	5.39 (5.05–5.66)	5.33 (4.94–5.72)	9.9 (6.8–13.3) μU/mL	11.4 (7.4–13.7) μU/mL	1.77 (0.53–3.10) mg/L	104 (66–136) mg/L
	1.03 (0.74–1.72)	1.19 (0.86–1.63)	5.43 ± 1.14	5.30 ± 1.03	1.37 ± 0.41	1.37 ± 0.41	3.47 ± 0.93	3.36 ± 0.98	5.00 (4.77–5.83)	5.00 (4.77–5.72)	9.1 (5.7–18.0) μU/mL	11.1 (9.0–14.7) μU/mL	1.48 (0.53–4.38) mg/L	105 (66–120) mg/L
Soerensen et al., 2014 (28)	0.76 ± 0.31	0.91 ± 0.31	4.26 ± 0.93	4.83 ± 1.12	1.35 ± 0.23	1.33 ± 0.19	2.56 ± 0.74	3.09 ± 0.97	5.35 ± 0.35	5.33 ± 0.38	24.1 ± 17.7 pmol/L	33.1 ± 23 pmol/L	NR	NR
	0.85 ± 0.31	0.83 ± 0.27	4.43 ± 1.12	4.84 ± 1.08	1.33 ± 0.19	1.28 ± 0.19	2.71 ± 0.74	3.18 ± 0.89	5.37 ± 0.26	5.3 ± 0.33	38 ± 21 pmol/L	15 ± 23 pmol/L	NR	NR
	0.78 ± 0.23	0.82 ± 0.23	4.23 ± 0.81	5.12 ± 0.93	1.3 ± 0.27	1.34 ± 0.27	2.57 ± 0.62	3.42 ± 0.97	5.41 ± 0.24	5.38 ± 0.24	27.5 ± 16.7 pmol/L	32.5 ± 16.8 pmol/L	NR	NR
Bellekçi-Koyu et al., 2019 (36)	2.09 (1.29–2.45)	1.72 (1.32–2.16)	6.31 (5.63–6.87)	5.75 (5.21–7.12)	1.17 (1.01–1.44)	1.19 (1.06–1.63)	4.00 (3.52–4.58)	3.73 (3.02–4.89)	5.83 (5.20–6.09)	5.58 (5.36–5.72)	15.94 (11.75–17.64) mU/L	13.64 (7.33–16.36) mU/L	NR	NR
	1.85 (1.43–2.49)	1.83 (1.32–2.67)	5.70 (5.17–6.45)	5.86 (5.15–6.22)	1.10 (0.89–1.45)	1.12 (0.93–1.50)	3.65 (2.96–4.60)	3.82 (3.00–4.34)	5.64 (5.39–5.96)	5.47 (5.42–6.46)	19.04 (18.09–25.49) mU/L	22.08 (15.05–28.54) mU/L	NR	NR
St-Onge et al., 2002 (31)	3.03 ± 1.52	3.03 ± 1.52	6.46 ± 1.43	6.51 ± 1.13	1.04 ± 0.29	1.05 ± 0.22	5.41 ± 1.45	5.43 ± 1.17	NR	NR	NR	NR	NR	NR
	2.87 ± 1.6	3.01 ± 1.3	6.08 ± 1.04	6 ± 1.25	1.01 ± 0.25	1.02 ± 0.22	5.06 ± 1.04	4.93 ± 1.22	NR	NR	NR	NR	NR	NR
Usinger L. et al., 2010 (27)	Study I: 1.04 ± 0.44; Study II: 0.88 ± 0.36	Study I: 1.08 ± 0.46; Study II: 0.99 ± 0.46	Study I: 5.3 ± 0.70; Study II: 5.2 ± 0.80	Study I: 5.2 ± 0.90; Study II: 5.2 ± 1.20	Study I: 1.3 ± 0.3; Study II: 1.3 ± 0.3	Study I: 1.3 ± 0.3; Study II: 1.3 ± 0.3	Study I: 3.5 ± 1; Study II: 2.5 ± 0.7	Study I: 3.3 ± 0.9; Study II: 3.3 ± 1.0	NR	NR	NR	NR	NR	NR
	Study I: 1.13 ± 0.40; Study II: 1.13 ± 0.40	Study I: 1.1 ± 0.58; Study II: 1.1 ± 0.58	Study I: 4.9 ± 0.70; Study II: 4.9 ± 0.70	Study I: 4.9 ± 0.80; Study II: 4.9 ± 0.80	Study I: 1.3 ± 0.3; Study II: 1.3 ± 0.3	Study I: 1.3 ± 0.3; Study II: 1.3 ± 0.3	Study I: 3.1 ± 0.7; Study II: 3.1 ± 0.7	Study I: 3.1 ± 0.7; Study II: 3.1 ± 0.7	NR	NR	NR	NR	NR	NR
Jenko Praznikar Z. el al. 2020 (29)	1.3 ± 0.6	1.4 ± 0.7	5.5 ± 1.6	5.2 ± 1.2	1.6 ± 0.5	1.5 ± 0.4	3.7 ± 1.5	3.5 ± 1.2	6.3 ± 0.9	5.7 ± 0.5	NR	NR	1.71 ± 1.09	1.86 ± 1.48
	1.4 ± 0.7	1.3 ± 0.7	5.6 ± 1.5	5.3 ± 1.2	1.6 ± 0.4	1.5 ± 0.4	3.8 ± 1.5	3.5 ± 1.2	6.1 ± 1.1	5.6 ± 0.6	NR	NR	1.78 ± 1.08	2.11 ± 1.66
Fathi Y. et al., 2017 (30)	145.3 (121.8; 168.8)	138.5 (120.5; 156.4)	212 (202.3–221.7)	183.3 (176.6–190.0)	45.4 (41.8; 49.0)	44.5 (43.3; 45.6)	132.3 (124.2; 140.4)	112.2 (107.3; 117.1)	NR	NR	NR	NR	NR	NR
	136 (120.7; 151.3)	132.7 (114.3; 151.1)	205.2 (195.6–214.7)	199.6 (192.7–206.4)	46.4 (43.4; 49.3)	42.7 (41.5; 43.9)	129.6 (122.0; 137.3)	127.6 (122.5; 132.6)	NR	NR	NR	NR	NR	NR
Agerholm-Larsen L. et al., 1999 (37)	1.65 ± 0.20	1.62	5.06 ± 0.22	4.8	1.3 ± 0.10	1.26	3.01 ± 0.2	2.81	NR	NR	NR	NR	NR	NR
	2.13 ± 0.53	1.82	5.01 ± 0.24	4.97	1.33 ± 0.18	1.29	2.81 ± 0.24	2.97	NR	NR	NR	NR	NR	NR
Nestel et al., 2013 (26)	1.35 ± 0.39	1.46 ± 0.19	5.30 ± 0.70	5.40 ± 0.29	1.30 ± 0.18	1.37 ± 0.08	3.40 ± 0.67	3.24 ± 0.24	NR	NR	NR	NR	NR	NR
	1.35 ± 0.39	1.37 ± 0.22	5.30 ± 0.70	5.00 ± 0.23	1.30 ± 0.18	1.40 ± 0.09	3.40 ± 0.67	3.30 ± 0.24	NR	NR	NR	NR	NR	NR
	136 (120.7; 151.3)	132.7 (114.3; 151.1)	205.2 (195.6–214.7)	199.6 (192.7–206.4)	46.4 (43.4; 49.3)	42.7 (41.5; 43.9)	129.6 (122.0; 137.3)	127.6 (122.5; 132.6)	NR	NR	NR	NR	NR	NR
Bellikci-Koyu et al., 2022 (25)	2.129 ± 0.069	2.369 ± 1.21	6.103 ± 1.05	5.94 ± 1.07	1.135 ± 0.227	1.146 ± 0.269	3.995 ± 0.89	3.768 ± 0.926	106.1 ± 10.9	107.5 ± 17.3	16.6 ± 8.7	16.4 ± 10.1	NR	NR
	2.05 ± 0.73	2.098 ± 0.99	5.984 ± 0.995	5.935 ± 0.923	1.19 ± 0.24	1.17 ± 0.23	3.85 ± 0.92	3.84 ± 0.85	101.3 ± 6.6	102.7 ± 9.9	18.7 ± 9.4	19.8 ± 10.3	NR	NR

	hs-CRP		IL-6		TNF-α		BP		BMI		Conclusion
Reference	BL	EL	BL	EL	BL	EL	BL	EL	BL	EL	
Richelsen et al., 1996 (32)	NR	NR	NR	NR	NR	NR	NR	NR	NR	NR	No significant differences in TGs, HDL-c between the I and C groups throughout the study period, even there was a reduction in total cholesterol and LDL levels initially, no significant long-term (6 months) difference seen between the groups.
	NR	NR	NR	NR	NR	NR	NR	NR	NR	NR	
Hepner et al., 1979 (33)	NR	NR	NR	NR	NR	NR	NR	NR	NR	NR	Yogurt supplementation of diet causes a significant reduction of serum cholesterol, without affecting serum TGs or weight. A similar effect was caused by milk in some of the studies, but the reduction in serum cholesterol caused by milk was statistically less significant when it did occur.
	NR	NR	NR	NR	NR	NR	NR	NR	NR	NR	
Sadrzadeh-Yeganeh et al., 2010 (35)	NR	NR	NR	NR	NR	NR	NR	NR	NR	NR	No differences were detected in the biochemical markers and blood count parameters among groups at baseline and during the intervention. A significant trial effect was detected in IL-6 levels. To 4 and 8 weeks, effect of the intervention on the % changes of the inflammatory and hemostatic markers, in the intention-to-treat population.
	NR	NR	NR	NR	NR	NR	NR	NR	NR	NR	
Antonopoulou et al., 2022 (34)			1.13 (0.77–1.52)	105 (84–131)	NR	NR	SBP: 130 ± 17; DBP: 77 ± 9	SBP: 123 ± 15; DBP: 73 ± 11	NR	NR	No differences were detected in the biochemical markers and blood count parameters among groups at baseline and during the intervention. A significant trial effect was detected in IL-6 levels. To 4 and 8 weeks, effect of the intervention on the % changes of the inflammatory and hemostatic markers, in the intention-to-treat population.
			0.80 (0.40–1.39)	102 (77–168)	NR	NR	SBP: 126 ± 18; DBP: 77 ± 9	SBP: 122 ± 16; DBP: 74 ± 10	NR	NR	
Soerensen et al., 2014 (28)	NR	NR	NR	NR	NR	NR	SBP: 127.1 ± 6.7; DBP: 72.2 ± 7	SBP: 122.8 ± 9.2; DBP: 70.8 ± 7	NR	NR	No significant change for TG, HDL-c blood pressure, blood glucose. Compared with the control diet, the cheese diet attenuated both total cholesterol and LDL-c. Insulin levels decreased seen after cheese consumption differed significantly from small increases observed after the control and milk period
	NR	NR	NR	NR	NR	NR	SBP: 122.3 ± 7.2; DBP: 68.9 ± 6.9	SBP: 121.6 ± 5.7; DBP: 71 ± 6.2	NR	NR	
	NR	NR	NR	NR	NR	NR	SBP: 124 ± 5.4; DBP: 71.3 ± 5.1	SBP: 123.6 ± 7.6; DBP: 72.2 ± 7.3	NR	NR	
Bellekçi-Koyu et al., 2019 (36)	0.22 (0.69–0.80)	0.16 (0.10–0.46)	15.82 (11.52–29.75)	13.47 (5.65–21.39)	12.01 (0.76–43.05) pg./mL	1.13 (0.49–8.33) pg./mL	SBP: 134.50 (115.25–140.50); DBP: 85 (77.50–92.00)	SBP: 118 (103.25–137.75); DBP: 78.5 (69.00–80.00)	30.67 (26.94–34.66)	30.58 (26.24–34.31)	Amelioration in TG, TC, HDL-c, LDL-c, in the intervention kefir group. The difference in fasting insulin from baseline to after intervention was significant in kefir group. TNF-α showed a significant decrease after the intervention of kefir. Blood pressure decreased significantly after the intervention in the kefir group, while only systolic blood pressure showed a modest decrease in the unfermented milk group.
	0.27 (0.21–0.41)	0.24 (0.13–0.50)	19.73 (13.85–28.71)	10.03 (6.16–16.45)	8.51 (0.49–25.85) pg./mL	4.12 (0.49–13.03) pg./mL	SBP: 132.5 (123.75–144.00); DBP: 89 (81.00–92.00)	SBP: 118 (105.75–137.00); DBP: 78.5 (66.75–89.50)	32.38 (29.18–34.59)	31.9 (29.05–33.71)	
St-Onge et al., 2002 (31)	NR	NR	NR	NR	NR	NR	NR	NR	NR	NR	Amelioration in TG, TC, HDL-c, LDL-c
	NR	NR	NR	NR	NR	NR	NR	NR	NR	NR	
Usinger L. et al, 2010 (27)	NR	NR	NR	NR	NR	NR	NR	NR	NR	NR	No significant effect
	NR	NR	NR	NR	NR	NR	NR	NR	NR	NR	
Jenko Praznikar Z, el al. 2020 (29)	NR	NR	NR	NR	NR	NR	NR	NR	NR	NR	a significant decrease in total Chol, LDL-c, and glucose after milk supplementation, whereas kefir supplementation resulted in decreased glucose and HDL concentrations.
	NR	NR	NR	NR	NR	NR	NR	NR	NR	NR	
Fathi Y, et al., 2017 (30)	NR	NR	NR	NR	NR	NR	NR	NR	NR	NR	No significant difference observed between kefir and control groups for TG, HDL-c. Significant reduction in kefir group for total cholesterol and LDL cholesterol levels compared to the control group
	NR	NR	NR	NR	NR	NR	NR	NR	NR	NR	
Agerholm-Larsen L, et al., 1999 (37)	NR	NR	NR	NR	NR	NR	NR	NR	NR	NR	After 8 weeks, there were no statistically significant between-group differences in TG levels, total cholesterol, in HDL-c. When comparing GAIO to the two placebo arms combined (including PP), GAIO had a significantly different change in LDL-c after adjusting for weight change.
	NR	NR	NR	NR	NR	NR	NR	NR	NR	NR	
Nestel et al., 2013 (26)	NR	NR	NR	NR	NR	NR	NR	NR	NR	NR	No statistically differences emerged between the full-fat fermented and not-fermented diets in any of the blood lipids variables measured.
	NR	NR	NR	NR	NR	NR	NR	NR	NR	NR	
	NR	NR	NR	NR	NR	NR	NR	NR	NR	NR	
Bellikci-Koyu et al., 2022 (25)	0.38 ± 0.42	0.32 ± 0.26	19.69 ± 14.34	15.03 ± 13.50	17.58 ± 18.87	10.57 ± 12.67	SBP: 128.9 ± 12.8; DBP: 83.9 ± 7.4	SBP: 120.0 ± 15.9; DBP: 79.5 ± 10.3	32.3 ± 5.8	32.5 ± 5.9	While no statistically differences emerged between groups according to blood lipid variables, blood pressure decreased significantly in both interventions. Kefir consumption resulted in inflammatory markers improvement, while control group obtained better outcomes in BMI.
	0.36 ± 0.27	0.35 ± 0.26	21.55 ± 15.49	16.75 ± 11.39	13.95 ± 14.90	10.61 ± 11.50	SBP: 131.9 ± 16.7; DBP: 85.3 ± 9.9	SBP: 123.0 ± 15.1; DBP: 81.5 ± 9.8	32.9 ± 5.4	32.7 ± 5.4	

Country: International alpha3 ISO code; Ind, Individuals; CVD, Cardiovascular Disease; pRCT, parallel Randomized Controlled Trial; Diet, Reported diet recommendations during intervention; HI, Healthy Individuals; I, Intervention group; C, Control/Placebo group; BL, Baseline; EL, Endline; ATB, Antibiotics; TG, TG (mmol/L); TC, Total Cholesterol (mmol/L); HDL-C, High Density Lipoprotein Cholesterol (mmol/L); LDL-C, Low Density Lipoprotein Cholesterol (mmol/L); Blood Glucose (mmol/L); Insulin (as reported in the paper); CRP, C-Reactive Protein (mg/L); hs-CRP, high sensitivity C-Reactive Protein (mg/L); IL-6, Interleukin 6 (pg/mL); TNF-α, Tumor necrosis factor alpha; BP, Blood Pressure (mmHg); SBP, Systolic blood Pressure; DBP, Diastolic Blood Pressure; BMI, Body Mass Index (kg/m^2^); NA, Not Applicable; NR, Not Reported; NS, Not Shown in the study, FQ: Frequency Questionnaire.

Despite applying our PICO criteria, the characteristics of the study populations naturally varied across the included studies. We carefully assessed each study to confirm that the participants were “healthy” adults aged 18 years or older. Although the definition of “healthy” was not always consistent, we checked registered study protocols, when available, to ensure alignment with our criteria. Importantly, we did not exclude studies involving individuals with elevated BMI or blood lipid levels, as we aimed to reflect the current demographic and metabolic profile of the general adult population.

Among the studies that investigated yogurt as an intervention, Nestel et al. (26) conducted a study in Australia involving 22 adults with overweight or obesity, with an average age of 60.5 years. Hepner et al. (33) conducted an RCT in the USA in 49 adults in good general health. Antonopoulou et al. (34) performed their study in Greece, including 58 healthy adults aged 35–65 years. In Iran, Sadrzadeh-Yeganeh et al. (35) conducted an RCT with 60 female volunteers aged 19–49 years, selected based on specific criteria: total cholesterol levels below 6.2 mmoL/L, TAG levels below 2.3 mmoL/L, and a BMI under 30 kg/m^2^.

Kefir studies also showed substantial variation. Bellekci-Koyu et al. (25, 36) included 62 patients with metabolic syndrome; Jenko-Praznkiar et al. (29) studied 28 middle-aged adults with overweight (average age ~ 50 years), Fathi et al. (30) evaluated 50 women with overweight or obesity, with an average age of 37 years; and St-Onge et al. (31) evaluated 13 mildly hypercholesterolemic males, otherwise healthy men.

All fermented milk studies were conducted in Denmark. Usinger et al. (27) studied 30 individuals with prehypertension or borderline hypertension. Richelsen et al. (32) included 87 healthy postmenopausal women. Agerholm-Larsen et al. (37), studied 26 obese but otherwise healthy adults. Finally, cheese was studied in 15 healthy subjects by Soerensen (28), also in Denmark.

Although studies meeting PICO criteria provide the highest level of evidence, our review, conducted following EFSA guidelines, our study protocol and Muka et al. (21), also included clinical trials meeting PIO criteria, i.e., trials where the conventional fermented dairy product was originally considered a control for their enriched or modified versions, and thus were examined as the intervention of interested without a direct comparator. It is important to note that we did not report the outcomes from the enriched or modified versions of the fermented dairy products. Instead, we focused solely on the results from the control or placebo groups consuming the plain, unfortified versions of the fermented dairy products, to maintain consistency and comparability across studies. The conclusions based on the findings from these studies have lower validity compared to PICO studies, as they are considered non-controlled studies.

Using this approach, we included 37 studies (Supplementary Table 5), primarily focused on yogurt (*n* = 25), but also cheese (*n* = 9), kefir (*n* = 2), and one study on koumiss. Similar to the PICO studies, the PIO studies included were conducted across a diverse range of countries. Denmark was the most represented (*n* = 4), followed by Greece (*n* = 3) and Finland (*n* = 3). Two studies were conducted in Canada, Estonia, Germany, Iran, Italy, Japan, South Korea, the Netherlands, Spain, and the USA. Several other countries were represented by a single study, underscoring the broad geographic interest of research on fermented dairy products and cardiometabolic health. In terms of sample size, most studies included fewer than 50 participants in the conventional fermented dairy products group, with many involving fewer than 20 participants. Both sexes were generally included in the study populations; however, some studies focused exclusively on specific subgroups, such as postmenopausal women. The average participant age was approximately 50 years. Consistent with our inclusion criteria, the study populations were generally healthy or mildly hypercholesterolemic, with the latter not receiving lipid-lowering medication. Participants were commonly instructed to maintain their habitual diets during the intervention period to ensure that any observed effects could be attributed specifically to the consumption of fermented dairy products.

##### 3.1.1.1 Findings from intervention studies on fermented dairy and cardiometabolic markers

###### 3.1.1.1.1 Yogurt

Nestel et al. (26) conducted a study in Australia involving 22 adults with overweight or obesity, with an average age of 60.5 years. This study reported no statistically significant differences between the full-fat fermented and non-fermented product in any of the blood lipid variables measured. Similarly, Hepner et al. (33) performed an RCT in the USA in 49 adults in good general health. They found that yogurt consumption did not significantly alter lipid levels; however, the control intervention (milk-supplemented diet) produced an increase in TG levels. In Greece, Antonopoulou et al. (34) carried out their study with 58 healthy adults aged 35–65 years. They observed no significant differences in biochemical markers between groups post intervention, although a reduction in IL-6 levels was noted in the yogurt group. In contrast, Sadrzadeh-Yeganeh et al. (35) conducted an RCT in Iran with 60 female volunteers aged 19–49 years, specifically selected based on criteria including total cholesterol levels below 6.2 mmoL/L, TAG levels below 2.3 mmoL/L, and a BMI under 30 kg/m^2^. This study found that the consumption of conventional yogurt significantly reduced total cholesterol compared to no consumption.

Additional evidence on the effects of yogurt on cardiometabolic markers comes from PIO studies. We identified 27 such trials and, in most cases (*n* = 21), no statistically significant changes were observed from baseline to post-intervention within groups, although some reported minor, non-clinically relevant changes (37–52).

However, six studies did report significant changes, though not always in a beneficial direction. For instance, Oosthuizen et al. (38) observed a decrease in HDL-c and an increase in the LDL-c/HDL-c ratio after 4 weeks of consuming 175 g/day of frozen yogurt in hyperlipidemic men. In contrast, Štšepetova et al. (53) in one of two separate trials, reported a reduction in non-HDL after 4 weeks of consuming 150 g/day of conventional yogurt containing 4.5 × 10^7^ CFU/mL *Streptococcus thermophilus* and 4.4 × 10^8^ CFU/mL *Lactobacillus delbrueckii* subsp. *Bulgaricus*; however, this effect was not replicated in the second trial. Sialvera et al. (39) found a reduction in LDL levels in patients with metabolic syndrome after 2 months of consuming a 100 g yogurt beverage twice daily, although no other cardiometabolic markers showed meaningful changes. Lastly, Nishiyama et al. (40) conducted a trial in individuals with slightly elevated blood lipids or glucose levels, who consumed 80 g/day of yogurt fermented with *L. bulgaricus*, *S. thermophilus*, and *L. acidophilus*. While this group showed only a statistical trend toward reduced total cholesterol (*p* = 0.06), a significant reduction in HbA1c was observed. When the analysis was repeated in a subgroup with borderline-high levels, the reduction in total cholesterol reached significance.

Although PIO studies provide valuable insights, their primary aim was not to assess the effects of conventional yogurt consumption *per se*. Consequently, statistical analyses were often focused on comparisons between the conventional yogurt and the enriched yogurt, rather than on the conventional yogurt independently. As a result, potentially relevant findings specific to conventional yogurt consumption may have been overlooked. Additionally, the considerable heterogeneity in study populations, intervention protocols, and durations may have influenced the outcomes and limited the comparability across studies. Nevertheless, given some evidence that conventional yogurt may exert beneficial effects on blood lipids, it should not be used solely as a control. Instead, it warrants investigation as a test food in its own right in well-controlled intervention studies.

###### 3.1.1.1.2 Kefir

Several studies have investigated the effects of kefir consumption on lipid levels, yielding mixed results. In their trial, Bellikci-Koyu et al. (25) conducted a study including 62 patients with metabolic syndrome. They reported that participants in the kefir intervention group experienced improvements in TGs, total cholesterol, HDL-c, and LDL-c levels. However, in another study, the same authors later found no significant changes in any of these lipid parameters following kefir (36) consumption. Jenko-Praznikar et al. (29) studied 28 middle-aged adults with overweight (average age ~ 50 years). They observed similar improvements in lipid levels, with both milk and kefir supplementation; however, kefir intake was associated with a decrease in HDL-c, suggesting a potential adverse effect in this regard. Fathi et al. (30) evaluated 50 women with overweight or obesity, with an average age of 37 years. They found significantly lower serum levels of total cholesterol and LDL-c in the kefir group compared to low fat dairy control, supporting a lipid-lowering effect. In contrast, St-Onge et al. (31) evaluated 13 mildly hypercholesterolemic males, otherwise healthy men. This study found no significant effects of kefir supplementation over 4 weeks on total cholesterol, LDL-c, HDL-c, triglycerides, or cholesterol fractional synthesis rates compared to milk.

Two additional PIO studies provided further insight. Yilmaz et al. (41) conducted an eight-week trial involving 13 dyslipidemic and 10 normolipidemic volunteers. They observed that kefir consumption significantly lowered total cholesterol and LDL-c levels in dyslipidemic participants, while no significant effects were seen in normolipidemic individuals. The TG levels remained unchanged, and a slight decrease in HDL-c was noted. In a single-blind, randomized crossover study, Bourrie et al. (42) compared the effects of consuming commercial versus pitched kefir in individuals with slightly elevated LDL-c. While neither kefir type significantly altered triglycerides, total cholesterol, or non-HDL cholesterol, pitched kefir led to a significant reduction in LDL compared to baseline, a benefit not observed with commercial kefir.

###### 3.1.1.1.3 Fermented milk

Usinger et al. (27) who studied 30 individuals with prehypertension or borderline hypertension, reported no significant effects of fermented milk consumption on cardiometabolic markers. Similarly, Richelsen et al. (32), studied 87 healthy postmenopausal women, found no significant differences between fermented and placebo milk groups in TGs, HDL-c, blood glucose, insulin levels, or blood pressure throughout the study. However, a transient reduction in LDL-c was observed in men at 3 months, which was not sustained until the end of the study at 6 months. In contrast, Agerholm-Larsen et al. (37), studied 26 participants with obesity but otherwise healthy adults, demonstrated a significant reduction in LDL-c after adjusting for weight change, concluding that GAIO®, a fermented milk product, may have a beneficial lowering effect in overweight and obese individuals.

Dönmez et al. (43) examined the effects of koumiss, a lightly alcoholic fermented mare’s milk beverage, on lipid levels in 18 sedentary male volunteers over 15 days. The study also evaluated the combined effect of exercise and koumiss consumption. While total cholesterol and TG levels tended to decrease and HDL-c levels tended to increase in both groups, these changes did not reach statistical significance (*p* > 0.05).

###### 3.1.1.1.4 Cheese

In the trial by Soerensen et al. (28), although no statistically significant changes were observed in cardiometabolic markers compared with the control diet, the cheese diet notably attenuated both total cholesterol and LDL-c levels. Additionally, insulin levels significantly decreased following cheese consumption, in contrast to the small, non-significant increases observed during the control and milk periods.

PIO studies on cheese consumption and its effects on blood lipids have produced mixed results. Some lacked sufficient statistical analysis of baseline and post-intervention data, particularly in the groups with conventional cheese [e.g., (44–46)]. While Raziani et al. (47) and Jauhiainen et al. (48) reported no significant effects on blood lipids, several other trials observed relevant changes. For instance, Santurino et al. (49) found that daily consumption of 60 g of commercial goat cheese in overweight or obese subjects did not significantly alter LDL-c but led to a significant improvement in the LDL-c/HDL-c ratio, an important marker of atherogenic risk. In a 12-week randomized parallel trial with a 2-week run-in period, Raziani et al. (50) compared regular-fat and reduced-fat cheese. No overall significant differences were observed in LDL particle concentrations between groups. However, a gender-stratified analysis revealed that men consuming regular-fat cheese showed decreases in total LDL-c and LDL particles, particularly medium-sized, along with reductions in LDL-c/HDL-c.

Conversely, Pintus et al. (51) reported that daily intake of 90 g cheese significantly increased HDL-c in individuals with mild hypercholesterolemia, although LDL and total cholesterol increased non-significantly. Finally, Davis et al. (52) in a four-month trial, found that daily consumption of 100 g of skim-milk mozzarella did not significantly alter blood lipid levels.

#### 3.1.2 Observational studies

To be able to represent all available human data, we have screened the observational studies as per our inclusion/exclusion criteria and included 17 papers. For clarity and ease of discussion, these have been organized into two main Supplementary Table 3 (cross-sectional studies) and Supplementary Table 4 (prospective longitudinal studies).

The cross-sectional studies (*n* = 5) primarily focused on yogurt and cheese consumption. The populations were drawn from a range of national cohorts, including major U.S. cohorts such as the Women’s Health Initiative and the Framingham Heart Study, specifically the Offspring (1998–2001) and Third Generation (2002–2005) cohorts. Other significant national cohorts included the Fifth Korean National Health and Nutrition Examination Survey (South Korea), the National Adult Nutrition Survey (Ireland), the ABCD_2 study (Italy), and a smaller Mexican cohort. Participant numbers varied widely, from 340 in the Mexican cohort to 35,352 in the Framingham cohort. These studies predominantly included the general adult populations, including postmenopausal women. Data on fermented dairy product consumption were obtained primarily through semi-quantitative Food Frequency Questionnaires (FFQs).

Supplementary Table 4 presents the prospective longitudinal data from 14 observational studies conducted across eight countries. These include Sweden (*n* = 3), Denmark (*n* = 2), and one study each from Australia, Brazil, Finland, France, Greece, Japan, and the Netherlands. Additionally, the Pan-European EPIC (European Prospective Investigation into Cancer and Nutrition) study, a large multicountry cohort, provided data from Denmark, Germany, Greece, Italy, New Zealand, Sweden, the United Kingdom, and France.

Participants were generally healthy and free from CVD at baseline. Sample sizes varied substantially, ranging from 1,746 participants in the MONICA cohort to 409,885 in the EPIC study. The minimum follow-up duration was 5 years, with some studies extending up to 30 years. While the majority of dietary assessments were conducted using validated semi-quantitative FFQs or 4-day food diaries (including one weekend day), the EPIC study uniquely employed 7-day weighed diet records for more precise dietary intake data.

Across these studies, the focus was primarily on the consumption of fermented dairy products, most commonly yogurt and cheese. Following this overview, the results have been summarized according to the specific type of fermented dairy product studied, highlighting a range of findings from observational studies, including null associations, protective effects, and sex-specific differences, reflecting the diverse methodologies and outcomes reported.

##### 3.1.2.1 Findings from observational studies on fermented dairy and cardiovascular outcomes

Several prospective cohort studies have assessed the associations between fermented dairy intake and lipid levels or CVD outcomes, using various definitions and methodologies. Guo et al. (54) calculated total fermented dairy by summing intakes of buttermilk, cheese, fruit yogurts, and soured milk products. Sellem et al. (55) included cheese, curd cheese, Petit-suisse, yogurts, and fermented milk under the category of fermented dairy, and provided stratified analyses by type (e.g., cheese vs. yogurt).

The method of assessing fermented dairy intake frequency varied across studies. For instance, Guo et al. (54) and Silva et al. (56) used energy-adjusted quartiles (g/week and g/day, respectively, with sex-specific adjustments in the latter), while Buziau et al. (10) applied tertile cut-offs based on residual energy-adjusted intakes. Patterson et al. (57) and Kouvari et al. (58) reported servings per day, while Zhang et al. (12) used a per 100 g/day increase model. Laursen et al. (59) used differences in daily servings, and Koskinen et al. (9) used intake ranges. Outcome endpoints across studies included CVD and CHD mortality; however, more specific outcomes such as ischemic heart disease (IHD) and total stroke were considered in Key et al. (60) and Laursen et al. (59), respectively.

In terms of findings, several studies reported null associations. Guo et al. (54) and Silva et al. (56) found no associations between total fermented dairy and CVD, CHD, or all-cause mortality. Similarly, no significant associations with individual fermented dairy types and cardiovascular outcomes were observed by Guo et al. (54). In Japanese adults, Lu et al. (61) also found no association between yogurt intake and mortality. Dalmeijer et al. (62) reported a non-significant trend toward reduced stroke risk with fermented dairy intake. Johansson et al. (63) found that greater intake of butter, fermented milk, and cheese tended to be associated with reduced risk of type 2 diabetes and myocardial infarction, although these findings did not always reach statistical significance.

Conversely, several studies did report protective associations. Sellem et al. (55) found that higher intake of fermented dairy was associated with reduced cerebrovascular disease risk. Zhang et al. (12) observed that fermented milk intake was significantly inversely associated with CVD and CVD mortality, after adjusting for sociodemographic and lifestyle confounders. Koskinen et al. (9) reported a 27% lower risk of CHD among individuals in the highest quartile of fermented dairy intake, after adjustment for potential confounders. Importantly, the manner in which fermented dairies are incorporated into the diet may affect outcomes. Laursen et al. (59) suggested that replacing semi-skimmed dairy with whole-fat fermented milk was associated with reduced ischemic stroke risk, regardless of fat content. Buziau et al. (10) found that high fermented dairy intake was associated with lower CVD risk only within certain dietary patterns.

Sex-specific associations were reported in some studies. Kouvari et al. (58) found that yogurt and cheese consumption was associated with reduced CVD risk, with yogurt having a stronger effect in women (20–30% lower risk per 200 g/day) and cheese showing modest benefit in men (5% lower risk per 30 g/day). In contrast, Patterson et al. (57) found only total cheese consumption to be inversely associated with MI risk, with no significant associations for other dairy products.

Cross-sectional studies generally reported weaker or inconsistent findings. Shi et al. (64) observed that higher intakes of total dairy, full-fat dairy, cheese, yogurt, and butter were associated with lower TG levels, with yogurt having the most pronounced effect (−5.4% per serving). Kim et al. (65) also noted a positive association between yogurt intake and HDL-c levels, and Wang et al. (66) found that yogurt consumers had lower TGs and systolic blood pressure. However, Salinas-Mandujano et al. (67) found no association between yogurt consumption and lipid markers, and Feeney et al. (46) observed no effects of cheese intake on metabolic markers, although high yogurt consumers exhibited lower anthropometric measures.

### 3.2 Risk of bias in human studies

**Figure 2:**
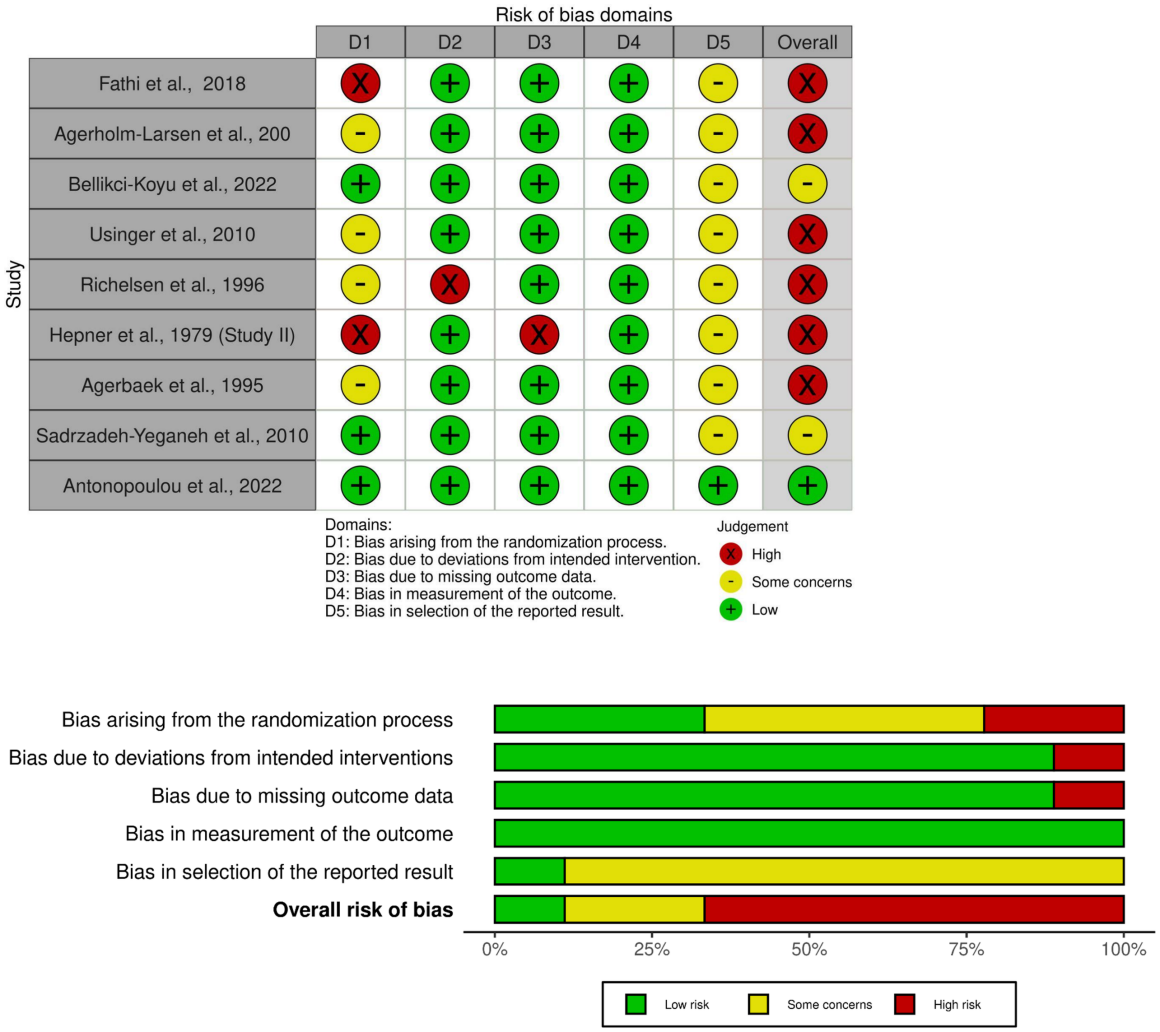
Risk of bias assessment for parallel trials.

Risk of bias was assessed only for studies meeting the PICO criteria. Among these, nine were parallel-group trials and five were crossover trials. In the overall assessment of the parallel-group trials (Figure 2), six of the nine studies were rated as having a high risk of bias (27, 30, 32, 33, 37, 68), two studies were judged to have some concerns (25, 35), and only one study was rated as low risk (34).

The domains primarily contributing to the high overall risk were the randomization process (absence of clear information) and the selection of the reported result. Eight of the nine studies raised some concerns regarding selective reporting, mainly due to the absence of or non-adherence to a pre-specified analysis plan. Bias due to deviations from intended interventions and missing outcome data was generally well controlled, with eight studies rated as having low risk in both domains. Notably, the measurement of outcomes was consistently rated as low risk across all nine studies, indicating reliable outcome assessment.

**Figure 3:**
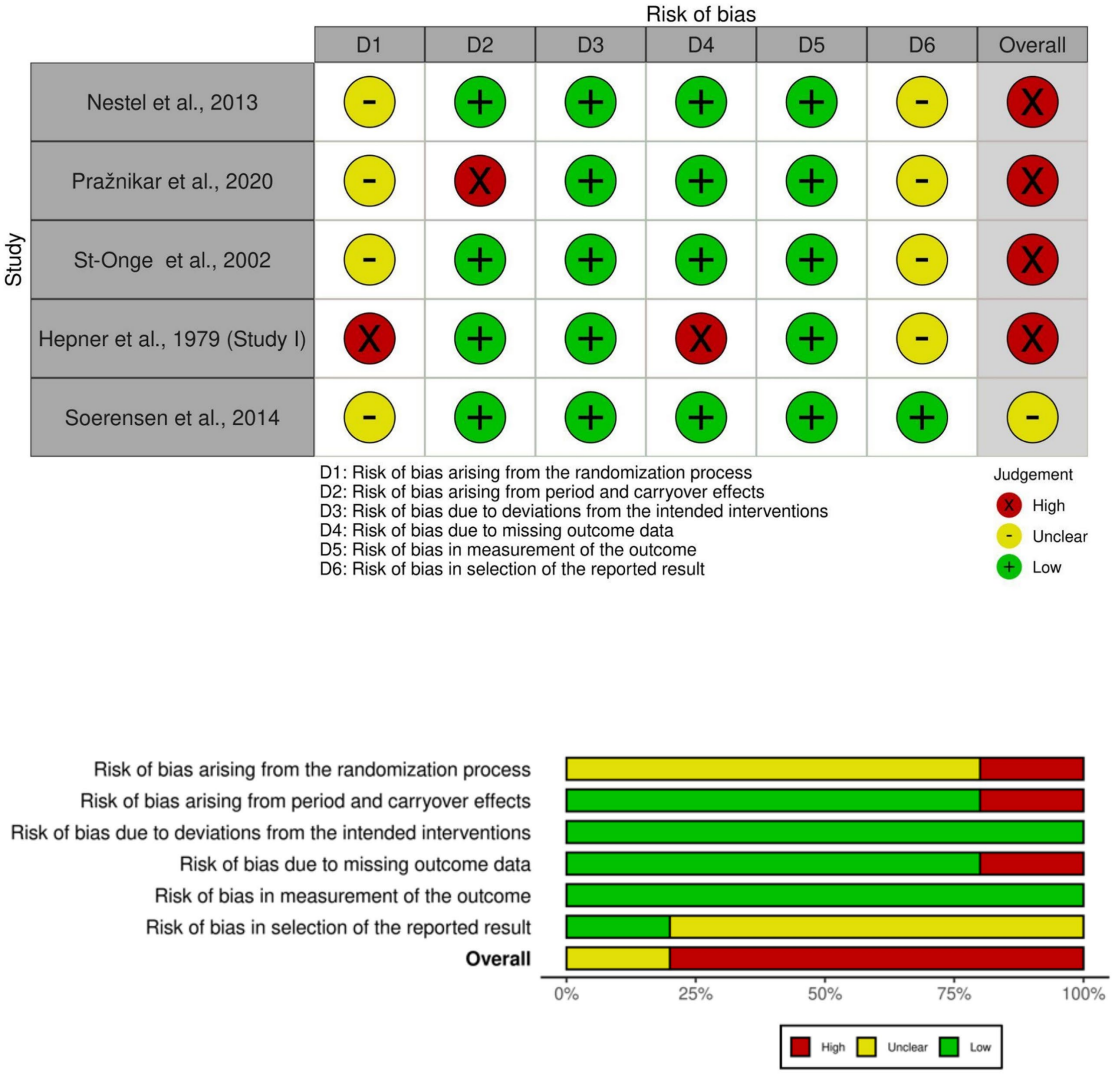
Risk of bias assessment for cross-over trials.

Five crossover trials were also assessed for risk of bias, incorporating an additional domain to assess bias arising from period and carryover effects. Four of the five trials were judged to have an overall high risk of bias (26, 29, 31, 33), while one raised some concerns (28). The randomization domain was the most problematic (e.g., the allocation sequence was not random), with four studies raising some concerns, and one study rated as high risk (33). Regarding period and carryover effects (Domain S), four studies were considered low risk, whereas one study (29) was rated high risk. Concerns regarding selective reporting were frequent and observed in four crossover trials. For missing outcome data, four studies were rated as low risk and one as high risk. Bias due to deviations from intended interventions and bias in the measurement of the outcome were consistently rated as low across all five studies (Figure 3).

These results indicate that while bias due to deviations from intended interventions and outcome measurement was generally well controlled in both parallel-group and crossover trials, issues related to the randomization process and selection of reported results were common. These methodological limitations underscore the need for improved rigor and transparency in the design and reporting of future trials.

### 3.3 Plausible mechanisms of action

Fermented dairy products may influence cardiovascular health through multiple interrelated mechanisms involving the dairy matrix, fermentation-derived microbial and biochemical processes, and host–microbe interactions.

The matrix of dairy products plays an important role in modulating their effects on lipid metabolism. For instance, cheese, due to its higher calcium and protein content, has been shown to attenuate the potential adverse effects of saturated fats found in dairy fat. When consumed in the form of cheese, dairy fat results in significantly lower LDL-c and total cholesterol levels compared to butterfat, suggesting that the food matrix itself has a significant effect on blood lipid profiles (46). Furthermore, the formation of calcium-fatty acid complexes and amorphous calcium phosphates in the duodenum is influenced by the dairy matrix, where fat in milk is present as small globules, while in cheese, fat is encapsulated by proteins such as casein. This difference in fat structure potentially facilitates a greater interaction between fat and calcium, leading to more efficient lipid metabolism (28, 69).

Fermentation processes in dairy products lead to the production of bioactive peptides and beneficial compounds that exert favorable effects on cardiovascular health. Fermented dairy products are rich in bioactive peptides, which are generated during the fermentation process and have been shown to exert antihypertensive and anti-inflammatory effects. These peptides can inhibit the angiotensin I-converting enzyme (ACE), thereby reducing blood pressure (11). Additionally, dairy products are rich in essential minerals, including calcium, potassium, and magnesium, which contribute to improved blood pressure regulation and overall cardiovascular health (11). In kefir, for example, bioactive peptides produced during fermentation may enhance the immune response by activating macrophages and increasing phagocytosis, which contributes to reduced inflammation and improved cardiovascular health (31). These peptides may also enhance insulin sensitivity and help regulate blood glucose levels, thereby indirectly reducing cardiovascular risk factors, such as metabolic syndrome. Fermented dairy, particularly kefir, may also contribute to the reduction of atherosclerosis-related inflammation. Studies indicate that kefir can lower the expression of cell adhesion molecules such as ICAM-1 and VCAM-1, which play key roles in the recruitment of monocytes during the early stages of atherosclerotic plaque development (42). This anti-inflammatory effect may be mediated by bioactive peptides, which can modulate immune cell activity and reduce chronic low-grade inflammation, a known risk factor for CVD (31).

The ability of probiotics to lower cholesterol can also be attributed to their production of short-chain fatty acids (SCFAs) during fermentation. SCFAs, including propionate and butyrate, can reduce cholesterol levels by blocking hepatic cholesterol synthesis and redirecting plasma cholesterol toward the liver. In addition, SCFAs can disrupt the enterohepatic circulation of bile acids by deconjugating bile salts, which further enhances cholesterol excretion (35). The presence of probiotics in yogurt (as detailed in Section 3.5) has also been linked to increased production of bile salt hydrolase, which facilitates the deconjugation of bile acids, promoting their excretion and contributing to cholesterol lowering (29).

Dairy consumption can also lead to an increase in SCFA production by gut microbiota, which plays a role in modulating cholesterol levels. Propionate, a predominant SCFA, has been shown to inhibit acetate’s cholesterol-generating effects, thereby reducing plasma cholesterol synthesis (70). However, studies suggest that kefir may not produce sufficient propionate to significantly affect cholesterol synthesis, indicating that the bacterial composition of fermented dairy products is an important factor in their hypocholesterolemic effects (25).

The cardiovascular health benefits of fermented dairy products are multifaceted, involving the dairy matrix, fermentation processes, probiotic strains, and bioactive components such as peptides, minerals, and SCFAs. The interaction between these factors contributes to the modulation of cholesterol levels, regulation of blood pressure, and reduction of inflammation, collectively supporting cardiovascular health.

### 3.4 Bioavailability of bioactive compounds

Bioavailability is a critical factor in determining whether bioactive compounds found in fermented dairy products can exert physiological effects. According to EFSA, bioavailability encompasses the processes of release from the food matrix (bio-accessibility), absorption, distribution, metabolism, and elimination. In the context of this review, the focus is on evaluating whether compounds such as peptides, probiotics, SCFAs, and minerals present in fermented dairy products are accessible and available in the human body to potentially modulate blood lipid levels.

#### 3.4.1 Peptides and protein-derived compounds

Several studies included in this review investigated products containing known bioactive peptides, particularly Val-Pro-Pro (VPP) and Ile-Pro-Pro (IPP). In the study by Usinger et al. (27), fermented milk containing VPP and IPP was consumed by participants with prehypertension or borderline hypertension. While these peptides have demonstrated *in vitro* activity, no significant differences in lipid outcomes were observed between the intervention and placebo groups, and plasma concentrations of the peptides were not measured. The authors acknowledged that the bioavailability of these tripeptides in humans remains uncertain, with previous studies reporting only modest increases in circulating levels. Similarly, the study by Hepner et al. (33) suggested a role for casein, the primary milk protein, in lipid regulation. Although casein-derived peptides were not directly analyzed, the authors proposed that peptide release during digestion could have contributed to the cholesterol-lowering effects observed with yogurt supplementation. However, no peptide profiling or absorption markers were included in the study.

#### 3.4.2 Probiotics

Several studies examined the presence of viable probiotic strains in fermented dairy products. In the study by Sadrzadeh-Yeganeh et al. (35), yogurt containing *L. delbrueckii* subsp. *bulgaricus* and *S. thermophilus* was associated with a reduction in total cholesterol as well as in total: HDL-c ratio. The authors proposed that components such as sphingolipids and milk fat may have contributed to these effects, though probiotic viability and bioavailability were not measured. Kefir-based interventions often included multiple microbial strains. Bellikci-Koyu et al. (36) reported a significant increase in the relative abundance of Actinobacteria and higher fecal detection of *Bifidobacterium* following kefir consumption, suggesting gastrointestinal survival of certain strains. However, systemic absorption or colonization was not assessed. Pražnikar et al. (29) found a significant reduction in serum zonulin levels, a marker of intestinal permeability after kefir supplementation, suggesting a functional effect on gut barrier integrity. The observed changes were attributed to the presence of specific probiotic bacteria and yeasts. Nonetheless, no measurements of microbial metabolites or systemic biomarkers were provided. In contrast, the study by St-Onge et al. (31) found increased fecal SCFA levels and bacterial counts following kefir intake, but no significant changes in blood lipids or cholesterol synthesis. The authors suggested that the bacterial dose may have been insufficient for measurable systemic effects.

#### 3.4.3 Short-chain fatty acids and lipid-related metabolites

SCFAs particularly propionate and butyrate, are microbial fermentation products proposed to influence lipid metabolism. While several studies mentioned SCFAs as potential mechanisms, only St-Onge et al. (31) measured fecal SCFA levels, which increased following kefir intake. However, this increase did not correspond to changes in plasma lipid levels, and no SCFAs were measured in circulation. Fathi et al. (30) discussed the potential role of SCFAs and bile salt hydrolase activity in lipid modulation following kefir consumption but did not measure these compounds or assess their systemic availability. The authors also cited calcium as a possible contributing factor, though the bioavailability of calcium was not directly evaluated.

#### 3.4.4 Minerals

Calcium was mentioned in several studies as a compound that may influence lipid absorption by forming insoluble complexes with fatty acids or bile acids. In the study by Soerensen et al. (28), participants consuming milk and cheese with higher calcium content showed increased fecal fat excretion and attenuated increases in LDL cholesterol compared to a control diet. Although no calcium absorption metrics were provided, the findings suggest that calcium delivered through the dairy matrix may influence lipid metabolism. Thorning et al. (45) also found higher fecal fat excretion after cheese consumption, implying that the dairy matrix may modulate fat and calcium absorption. The results suggest matrix-related effects on nutrient bioaccessibility rather than direct bioavailability data.

Across the included studies, evidence for the bioavailability of bioactive compounds from fermented dairy products remains indirect. No human study included in this review measured systemic concentrations of peptides, probiotic strains, or microbial metabolites. Observed physiological effects are often attributed to bioactive compounds based on assumptions about their presence, prior literature, or theoretical mechanisms rather than direct evidence of absorption or activity in the human body.

Based on the human studies reviewed, the evidence supporting the bioavailability of bioactive compounds in fermented dairy products is limited. While certain compounds, such as calcium and probiotic strains, show plausible functional effects, the lack of direct measurements of digestion, absorption, or systemic concentrations precludes definitive conclusions.

### 3.5 Characteristics of the dairy fermented foods

The definition of fermented milk (CAC/RCP 243, 2003) and cheese (CAC/RCP 283, 1978) have been clearly made by Codex Alimentarius. As shown in Table 2, yogurt and yogurt-based products (including yogurt drinks/beverages, smoothies and frozen yogurt) were the most frequently studied fermented milk products, examined in 30 of the included studies. Additional products evaluated (Table 3) comprised kefir (seven studies), cheese (10 studies) and other types of fermented milks (four studies). Most of the products used were either commercially available items (26 studies) or their source was unspecified (19 studies). A smaller number were developed in laboratory settings, more specifically three yogurts (71–73) and two kefir products (36, 42), while one study investigated a traditional homemade fermented milk product (koumiss) (43).

For yogurts and yogurt-based products, the most common starter cultures reported in the included studies were the protocooperation mix of *S. thermophilus* and *L. delbrueckii* subsp. *bulgaricus*, which are well specified by Codex Alimentarius Standard No. 243/2003. Alternative culture yogurts made by the starter culture of *S. thermophilus* and other *Lactobacillus* species were reported, including *L. acidophilus* (37, 40, 71, 74, 75), *Lacticaseibacillus rhamnosus* (37) and *Lactiplantibacillus plantarum* (76). In a few studies, *Bifidobacterium infantis* (75), *B. lactis* (72, 74), and *Enterococcus faecium* (37) were used. Kefir products were produced using defined starter cultures prepared from kefir grains (25, 36, 42), as indicated by Codex Alimentarius Standard No. 243/2003. They consisted mostly of species of the genera *Leuconostoc* and *Lactococcus, L. kefiri*, along with lactose-fermenting yeast (*Kluyveromyces marxianus*) and non-lactose-fermenting yeast (*Saccharomyces unisporus*). The starter cultures used for pitched kefir were previously isolated from traditional kefir grains, which naturally exhibit variations in the microbial composition and diversity (42). In addition to the above, the latter also included *L. kefiranofaciens*, *Acetobacter pasteurianus* and more stains of yeasts (*Pichia fermentans*, *S. cerevisiae*, *Kazachstania unispora*). No starter culture details were reported for cheese, except for one study where they did not add any starter cultures (51).

Despite the significant role of the origin of milk on the nutritional value and the organoleptic properties of fermented dairy products (77, 78), most of the included studies (43 out of 51) did not include this information; however, some studies reported it. Four yogurt samples (53, 73, 76, 79) one kefir sample (29) and one cheese sample (28) were made from cow’s milk, one yogurt sample (73) and one cheese sample (51) from ewe’s milk, and one cheese sample from goat’s milk (49).

When available, the manufacturing process of the fermented milk products involved the typical stages: pasteurization of milk (80–115 °C, 5–60 min), cooling at temperature optimum for fermentation (25–50 °C) depending on the type of microorganisms involved (e.g., mesophilic or thermophilic cultures), the addition of specific starter culture, incubation at an optimum temperature to control fermentation (35–42 °C, 7–24 h) resulting in acidification (pH of 4.2–4.7), with or without milk protein coagulation, pasteurization (optional), cooling (23–27 °C), packing and cold storage (2–6 °C). In the case of frozen yogurt production, post-fermentation addition of flavorings, stabilizers/emulsifiers and/or sugars is usually included, followed by freezing the mixture (78). The cheese-making process involves the following main general steps for all varieties of cheese: pasteurization of raw milk (optional), acidification by indigenous lactic acid bacteria, ‘backsloping’ culture or starter culture, acid or enzymatic (rennet) coagulation, optional post-coagulation processes (i.e., cutting, cooking (scalding), cheddaring, curd washing, stretching, molding/drainage, pressing, salting), ripening (maturation, optional), packing, storage. In two studies, reconstitution of milk by adding skimmed milk powder (9–12%) and/or gelatin (0.4% w/v) in water was reported to produce yogurt (71) and fermented milk product Gaio® (27). Additionally, in some cases, the fresh whole milk was subjected to a skimming process (33, 38, 52, 73, 79) or was supplemented with skimmed milk powder (75). In some cases, it was reported that regulatory production standards were followed, including French regulations for yogurt production (80) and PDO guidelines for ‘Queso de Murcia’ cheese production (49).

Out of the 51 included studies, 32 studies reported the quantitative determination of proximate composition, predominantly for yogurt, including moisture, total fat, total carbohydrate, crude protein, ash, dietary fiber, and energy. These reported values meet the required specifications for milk protein (min. 2.7%) and fat (less than 10% for fermented milk, kefir and koumiss and 15% for yogurt and alternate culture yogurt), as set by the Codex Alimentarius (CAC/RCP 243, 2003). Also, this standard stipulates a minimum of 0.6% acidity (lactic acid), a minimum of 10^7^ colony-forming units (CFU)/g of microorganisms (total microorganisms in the starter culture) and a minimum of 10^6^ (CFU)/g of labelled microorganisms. Notably, findings suggest that certain fermented dairy products may qualify for health-related nutrition claims; 17 yogurt-based products reported fat contents below the thresholds defined in Regulation (EC) No 1924/2006 for “low-fat” nutrition claims (i.e., ≤3 g/100 g for solid foods and ≤1.5 g/100 mL for liquids), indicating their potential eligibility for such labelling under EU regulations. Furthermore, four yogurts had saturated fatty acid content not exceeding the maximum of 1.5 g/100 g, meeting the criteria for a “low-saturated fat” claim. One kefir could meet the requirements for “low sugars” claim (i.e., ≤5 g/100 g for solids or ≤2.5 g/100 mL for liquids).

Despite the relevance of identifying and quantifying bioactive compounds, including peptides, exopolysaccharides, unsaturated lipids, vitamins and minerals, very few included studies addressed these issues. As reported in the study of Olmedilla-Alonso et al. (73), low-fat and whole ewe’s milk yogurts were richer in SCFA than whole cow’s milk yogurt (20.26–21.17 vs. 11.31 g/100 g fat). Both ewe’s milk yogurts had similar content of PUFA (2.45–2.75 g/100 g fat) and CLA (0.24–0.27 g/100 g fat) with those of cow’s milk yogurt, but a two-fold higher *n*-3 *α*-linolenic acid content (0.76–0.87 g/100 g fat) and a lower *n*-6: *n*-3 ratio (2.07–2.13). Data for minerals (calcium, magnesium and potassium) showed that ewe’s milk yogurts contained higher amounts of calcium (2012.2–2063.1 mg/kg) and magnesium (171.1–171.8 mg/kg) but lower amount of potassium (1243.0–1264.3 mg/kg) compared with the respective values for cow’s milk yogurt (1081.3, 84.3 and 1,380 g/kg, respectively). Calcium was reported in similar or even higher amounts in cheese samples (1,600 mg/kg for skimmed cow’s milk cheese, Gamalost® and 8,000 mg/kg for the Gouda-type cheese, Norvegia®) of the included studies (44) but lower amounts (~1,200 mg/kg) in kefir samples (29, 30). Magnesium and potassium were reported for Gamalost® (130 and 980 mg/kg) and Norvegia® (330 and 770 mg/kg) (44). Sodium content varied between fermented dairy products; it was reported to be 396 mg/kg of yogurt smoothie (72), 1,000 mg/kg of low-fat plain yogurt (34, 81), 240 mg/kg for Gamalost® and 4,020 mg/kg for Norvegia®. Between regular-fat and reduced-fat Danbo and cheddar cheeses higher contents of SFA, MUFA and PUFA were observed (16.75–20.25 vs. 8.25–14.00, 7.00–11.50 vs. 3.25–8.25 and 0.75–1.75 vs. 0.25–0.75 g/100 g cheese, respectively), possibly reflecting their difference in total fat content (47, 50). The four cheeses had similar levels of calcium and sodium (667.5–772.5 and 1500.0–1750.0 mg/100 g, respectively). Values for phenolic compounds and vitamins were rarely reported in the included studies. Total phenolic content of 2.1 mg/g dry weight was estimated in low-fat plain yogurt (81). In the study of Richelsen et al. (32), the content of vitamin E and C was reported in the fermented milk product Gaio® at concentrations of 5 mg/kg and 100 mg/kg, respectively. Peptides were not investigated extensively in the reviewed studies; Usinger et al. (27) stated that 100 mL of the fermented milk product Cardio04® contained 470 mg total peptides, 2.5 mg VPP and 1.1 mg IPP. In particular studies, attention was focused on cholesterol contents in yogurt (30–40 mg/kg or 50 mg/L) (37, 82), yogurt smoothie (44 mg/kg) (72), yogurt drink (50 mg/kg) (83), kefir (62 mg/L) (31), mozzarella cheese (600 mg/kg) (52) and the fermented milk product Gaio® (50 mg/kg) (32). Compositional data for some cheese samples were reported through detailed descriptions of their contribution to the macro-and micronutrients and key bioactive compounds of the intervention diets (28, 45, 46).

Quantification of the microbial populations in the final products was reported in 9 studies on yogurt (35, 37, 53, 71, 74, 75, 79, 80, 84), 1 study on yogurt smoothie (72), 3 studies on kefir (25, 41, 42) and 2 studies on the fermented milk product, Gaio® (32, 68). These studies demonstrated that the freshly prepared products typically contained between 10^7^ and 10^10^ CFU/g of starter culture microorganisms. Only a handful of studies examined the microbial stability of the product. In two studies on yogurt (71, 74), as well as in one on the fermented milk product Gaio® (68), the authors stated that bacteria count in the final product remained almost stable during 1-week storage at 5–7 °C. Georgakouli et al. (79) investigated the population of the added starter cultures (*S. thermophilus* and *L. bulgaricus*) and yeasts/molds throughout storage of yogurt for 2 months at 4 °C. The results differentiated between the two bacteria; population of *S. thermophilus* remained somewhat stable for 30 days (~9 log CFU/g) and then a drop of ~1 log was observed at the end of storage, whereas *L. bulgaricus* counts tended to slightly increase for 1 week (6–6.5 log CFU/g) and after 10 days a reduction was noticed to 4.5 log CFU/g. The latter generally attained a lower population during fermentation and storage compared to the former. It is important to note that in some mild acidifying yogurts, *L. bulgaricus* may be present but does not grow appreciably, and its metabolic activity can be reduced or absent (85). Although this feature is desirable in such yogurt types, it may affect the metabolome and nutritional properties of the final product (85). On the other hand, yeasts and molds presented the opposite trend and grew during storage from 1.5 to 7 log CFU/g.

A notable limitation observed in some included studies was the reliance on prior published data on compositional characteristics of the fermented dairy products used, which may introduce measurement bias. In the study of Santurino et al. (49), the physicochemical composition of goat cheese samples was not directly measured but inferred from earlier work (86). Also, the fatty acid composition of two cheeses samples were either supplied from the manufacturer (52) or in the case of Pintus et al. (51) inferred from previous publication (87). Oosthuizen et al. (38) obtained the fatty acid composition data for frozen yogurt from South Africa Food Composition Tables. Pražnikar et al. (29) described the composition of the microbial population of kefir based on previous research by Vardjian et al. (88). Similarly, in the study of Richelsen et al. (32), the microbial counts were inferred from previous investigations. The latter approach may be justified when the nutrients of interest are well-characterized and stable. For example, the study by Vardjian et al. (88) reported, demonstrated high microbial stability, as well as similar counts and composition of lactobacilli and yeasts in kefir grains and beverage, over 10 weeks of propagation across two separate laboratories, which yielded comparable results. Their findings support the view that a stable microbiome of kefir grains ensures reproducible and constant product quality. Also, compositional data for some cheese samples were reported through detailed descriptions of their contribution to the macro-and micronutrients and key bioactive compounds of the intervention diets (28, 45, 46).

In the included studies, despite the complex and diverse microbiota of fermented dairy products-particularly kefir, which is traditionally produced using kefir grains-detailed compositional characterization was largely lacking. Previous research has shown that the microbial composition of kefir can vary significantly depending on factors such as the type of milk used, geographical origin, and fermentation conditions (89, 90). Among the studies reviewed, only one employed quantitative Real-Time Polymerase Chain Reaction for microbial profiling of kefir (41), and only one reported species-level identification (29). As most products evaluated were commercially available, sensory acceptability was presumed; however, no specific sensory data were provided, except for a brief description of the organoleptic properties of a cheese product (49).

The insufficient reporting of batch-to-batch variation and the variability in analytical techniques in the included studies highlight the fact that rational evaluation and control of product quality consistency are essential to ensure efficacy and safety. Without sufficient data on batch-to-batch variation, a significant gap remains, particularly in studies that reused the same products by referencing earlier research. Moreover, compositional characterization is often lacking in the existing literature. Therefore, this review highlights the importance of addressing these issues in future clinical trials to ensure reproducibility and accurate interpretation of results.

This comprehensive approach enabled us to uncover the “tip of the iceberg” regarding the current state of knowledge in this field. More specifically, although insights from the literature allowed us to identify key knowledge gaps in product characterization, as reflected in Tables 1, 2, our synthesis reveals a critical limitation in the field: a lack of standardized, comprehensive profiling of fermented dairy products used in clinical and observational studies. Despite their long history of consumption and cultural relevance, many of the fermented dairy foods included in our review were insufficiently characterized in terms of microbial composition, biochemical properties, and production parameters.

To advance the field and enhance scientific rigor in future studies on fermented dairy products, we suggest implementing comprehensive product characterization and study design. This should include specifying the origin and type of milk, explicitly listing the starter cultures, identifying microbial species and strains (e.g., through metagenomic approaches), as well as quantifying viable counts both at production and at the end of shelf-life. Furthermore, studies should analyze and provide detailed report of macronutrients (e.g., fat, protein, carbohydrate), micronutrient (e.g., sodium, calcium, magnesium), and key bioactive components (e.g., SCFAs, peptides, CLA, PUFA, vitamins) for the final products using validated analytical methods. Clear documentation of processing parameters (e.g., pasteurization parameters, fermentation time/temperature, and storage conditions), along with assessment of batch-to-batch variation, particularly microbial stability, is essential to ensure reproducibility. These recommendations align with the EFSA guidance for substantiating health claims and incorporating such standardized information in future studies will enable more meaningful comparisons and support the identification of bioactive components driving cardiovascular effects.

### 3.6 Relationship between consumption of the fermented food and functional effect

As outlined above, the primary aim of our systematic review is to evaluate whether consumption of fermented dairy foods confers potential health benefits on blood lipid profiles in healthy adults. To address this, we have detailed relevant studies and their outcomes in the section titled “*Identification of pertinent human efficacy studies*” Both sections have been organized in accordance with EFSA guidelines to ensure clarity and consistency. To avoid redundancy, we do not reiterate detailed descriptions of the individual studies here. Instead, this section focuses on providing targeted, concise information aligned with EFSA criteria, as applied in the PIMENTO WG3 framework for structuring our manuscripts.

#### 3.6.1 Substantiation of causal relationship

Evidence from RCTs and observational studies suggests that certain fermented dairy products (e.g., yogurt, kefir, cheese, fermented milk) may specifically affect blood lipid markers, particularly LDL-c, total cholesterol, and HDL-c. However, the effect is not uniformly observed across all products or studies, and some beneficial effects (e.g., on inflammation) may occur independently of lipid changes, suggesting a partially specific effect linked to product type and microbial content. Furthermore, a consistent dose–response relationship has not been clearly demonstrated. Some studies report effects at 80–175 g/day of yogurt or kefir, while others show no effect at similar or higher doses. The lack of standardized dosing and variability in microbial content hinders robust dose–response conclusions. Where observed, reductions in LDL-c or total cholesterol were modest but could be physiologically relevant when accumulated over time (e.g., 5–10% reductions). In some kefir and yogurt trials, LDL-c reduction ranged between 0.2–0.5 mmol/L. However, these effects were not consistently replicated, limiting confidence in the magnitude.

Intervention durations ranged from 2 to 24 weeks, with a median of 4–5 weeks. This is generally aligned with the expected time for dietary interventions to affect lipid metabolism as indicated by EFSA (91) although some effects, especially related to gut microbiota modulation, may require longer exposure to manifest fully.

The effects observed across studies were partially consistent but largely heterogeneous. While some RCTs (30, 35, 40) reported significant improvements in lipid profiles, others (27, 31) found no measurable effect. These inconsistencies can be attributed to several factors, including variations in microbial strains, differences in product composition, heterogeneity in study populations, and limited sample sizes. Despite this variability, trends indicating potential lipid-lowering benefits were more frequently observed with kefir and certain types of yogurts.

#### 3.6.2 Characterization of the relationship

Effects were demonstrated in healthy adults aged 18–70, including individuals with mildly elevated cholesterol or metabolic syndrome. Some studies included subgroups such as postmenopausal women [e.g., (32)] or overweight individuals, which are reflective of the target population for cardiovascular risk prevention. The majority of studies were conducted under free-living conditions with participants consuming the fermented dairy products as part of their usual diet. A few studies included more controlled settings (e.g., food provision or metabolic measurements), but these remained consistent with real-world consumption scenarios.

Limited evidence is available on long-term sustainability, as most studies were of short duration. However, some studies [e.g., (63, 66)] suggest that beneficial effects can persist over several weeks. Observational studies with long-term follow-up (up to 30 years) support a potential sustained protective association. Effects on LDL-c or total cholesterol were observed with daily intakes as low as 80–100 g of yogurt or 100 mL of fermented milk. Some kefir trials showed benefit with ~200 mL/day. However, dose–response thresholds remain unclear due to inconsistent methodologies.

The effective doses observed in studies are generally within the range of habitual intake in many European and Asian populations (e.g., ~1–2 servings/day). Therefore, these quantities are realistically achievable as part of a balanced diet and align with national dietary guidelines.

In conclusion, while the current body of evidence suggests a plausible relationship between fermented dairy consumption and improved blood lipid profiles, especially reductions in LDL-c, the heterogeneity in findings, product formulations, and study quality limit the ability to draw definitive causal conclusions. Further standardized trials are warranted to confirm specific effects, establish dose thresholds, and assess sustainability over time.

### 3.7 Safety

Twelve of the 51 included studies (24%) explicitly reported at least one adverse effect associated with the consumption of yogurt (34, 83, 92–94), kefir (30, 31, 36), cheese (48, 49), and other fermented milk products (27, 68).

Most of the reported adverse effects were mild, self-limited gastrointestinal symptoms. The most frequently reported side-effects were gastrointestinal complaints (borborygmi, loose stools, obstipation) (27, 34, 68, 83, 92), abdominal bloating, cramping and/or distension (25, 27, 30, 34, 48), nausea, vomiting, diarrhea, halitosis, and/or constipation (25, 31, 48, 49, 94). Severe bloating was reported only in the study of Fathi et al. (30) and led to the discontinuation of intervention in 17 participants before the first follow-up visit. Only one study of Jauhiainen et al. (48) mentioned adverse effects, headache and tiredness. No gastrointestinal side effects or systemic reactions were reported, and no participant withdrawals were attributed to safety-related concerns for the rest of the studies (39 studies) (26, 28–30, 32, 33, 35–47, 50–53, 71–76, 79–82, 84, 95–99).

The self-limited gastrointestinal symptoms likely align with the intake of live microbial cultures and fermentation by-products (e.g., short-chain fatty acids, carbon dioxide) formed during bacterial metabolism. These effects are generally dose-dependent and appear to reflect an adaptive colonization phase by microorganisms (probiotic organisms) rather than intrinsic toxicity (25, 29–31, 36). However, no information concerning the association between the fermentation process and the reported adverse effects was provided in the studies included in this review. Also, the vast majority of the included studies implicitly specified subgroups recommended avoiding the consumption of fermented dairy products by excluding participants with a self-reported history of lactose intolerance, allergy or other adverse effects to the dairy products (30, 51, 84, 93).

Only one of the 51 studies (2%) cautioned against excessive intake of whole ewe’s milk yogurt, stating that “*The energy value of whole ewe’s milk yogurt may be a problem if the energy content of the diet is not controlled*” (73). No other studies issued overconsumption warnings.

No additional restrictions related to the safety of dairy-fermented foods were reported in any of the reviewed studies. Concerning the overall evidence qualification, given the consistent demonstration of only mild, self-limited gastrointestinal effects in less than half of the studies, together with the large proportion of studies reporting no safety concerns, the safety evidence of fermented dairy products in healthy adults is reasonably evaluated as convincing and sufficient (22).

## 4 Conclusion and summary of the evidence

The focus of this review lies in evaluating the effects of conventional dairy fermented products on blood lipid profiles, specifically cholesterol, TG, HDL-c, and LDL-c levels, in individuals without pre-existing health conditions. It is important to note that the definition of “healthy” individuals varied across studies. Nonetheless, common inclusion criteria generally involved the absence of medication use (particularly those affecting lipid metabolism) and the lack of diagnosed medical conditions.

The modulation of lipid metabolism by fermented dairy products does not rely solely on the essentiality of nutrients, but rather on the bioactive components formed during fermentation, such as SCFAs and peptides and probiotic cultures. These constituents have been shown to influence lipid absorption, bile acid metabolism, and systemic inflammation—mechanisms that may contribute to favorable changes in blood lipid levels.

Importantly, our review synthesizes the available evidence in accordance with EFSA guidance for the substantiation of evidence-based potential health benefits. We critically evaluated the quality, consistency, and relevance of the data, alongside the appropriateness of study designs and selected endpoints. Each part of the results and discussion section was assessed in line with the corresponding EFSA evaluation criteria.

In the section titled *“Identification of pertinent human efficacy studies,”* our assessment concluded that the current body of evidence is “*neither convincing nor sufficient.”* This evaluation was primarily driven by the absence of positive results in most studies, considerable inconsistencies in study design, limited investigation into underlying mechanisms of action, and inadequate characterization of the fermented dairy products examined in PICO and PIO studies.

The bias assessment of the PICO studies included was also rated as “neither convincing nor sufficient” because many trials were at “high risk” of bias. Of the nine parallel-group trials included, only one was assessed as having a low risk of bias, whereas none of the five crossover trials were at low risk. These weaknesses, particularly related to inadequate reporting of the randomization process and selective reporting of results, lower confidence in the evidence. Although bias due to deviations from intended interventions and missing outcome data was generally well controlled, the high overall risk of bias introduces uncertainty regarding the reliability of the observed effects and may skew the conclusions from individual studies and systematic reviews with meta-analyses when combined. This limitation underscores the need for future randomized trials to adopt more rigorous design and reporting practices, including transparent randomization procedures and adherence to pre-registered protocols. Strengthening methodological quality is essential to enhance the reliability and applicability of the findings in this field.

Our evaluation of the sections “Characterization of the fermented foods” and “Bioavailability of bioactive compounds” resulted in a “neither convincing nor sufficient” rating. Many studies lacked reporting on batch-to-batch variation, and there was considerable variability in analytical methods used to characterize products. These shortcomings underscore the need for rigorous product quality control and standardized reporting to ensure the reliability of findings. Compositional analysis, compliance with GMP/HACCP standards, shelf-life data, and storage conditions were frequently absent or inadequately described.

Likewise, very few studies were designed to explore or validate mechanisms of action. Although some offered mechanistic insights, such instances were rare. For example, SCFAs may lower cholesterol by inhibiting hepatic cholesterol synthesis and promoting cholesterol clearance via bile acid excretion. Propionate, in particular, has been shown to counteract acetate-driven cholesterol production, thereby reducing plasma cholesterol synthesis (70). However, studies suggest that kefir may produce insufficient propionate to exert meaningful effects, highlighting the importance of microbial composition in determining functional outcomes (25). Furthermore, probiotic strains in yogurt have been associated with increased bile salt hydrolase activity, facilitating bile acid deconjugation and excretion. While this body of literature is growing, these mechanisms were rarely the primary focus of the studies included in this review. Thus, we rated this domain as “neither convincing nor sufficient.”

Regarding safety, our evaluation was “no or very limited evidence.” Although some trials reported adverse effects, comprehensive safety assessments, such as pathogens, were generally lacking. Information on tolerance, long-term safety, and adverse event monitoring was either absent or insufficiently reported.

Taken together, these findings indicate that while the lipid-modulating effects of fermented dairy products may extend beyond the provision of essential nutrients, substantiating their benefits requires more rigorously designed human intervention studies. Such studies, with a proper control, must consistently demonstrate reproducible effects on lipid markers such as reductions in total cholesterol, LDL-c, or TGs, and/or increases in HDL-c, following consumption of specific products like yogurt, kefir, or cheese. A proper control would be a non-fermented dairy product that is matched in energy, macronutrients, and micronutrients (e.g., calcium, vitamin D) to the fermented version, to help isolate the effects attributable specifically to fermentation. For instance, pasteurized milk (when comparing to fermented milk or kefir), heat-treated yogurt base without live cultures (when comparing to probiotic yogurt), or unfermented cheese curd or dairy blends (when comparing to ripened cheeses) could be chosen as comparators. This design would help control for the effects of the dairy matrix itself, allowing researchers to isolate the contribution of fermentation-derived bioactive compounds and to test mechanistic hypotheses, such as the roles of SCFA production, bile salt hydrolase activity, and other fermentation-specific pathways, in lipid modulation.

In conclusion, this systematic review found that current evidence does not support the lipid-modulating effects of conventional fermented dairy products in healthy individuals, due to a lack of positive results, considerable inconsistencies in study design, high risk of bias, and inadequate product characterization. Mechanistic data and safety reporting were also limited, further weakening the strength of the evidence. To advance the field, rigorously controlled human trials using well-characterized products and appropriate comparators are needed to determine whether conventional fermented dairy products have any lipid-lowering effects.

## Data Availability

The original contributions presented in the study are included in the article/Supplementary material, further inquiries can be directed to the corresponding authors.
