# Supplementary material for: Fermented dairy product consumption and blood lipid levels in healthy adults: a systematic review
Source: Front Nutr. 2025 Sep 11;12:1651134. doi: 10.3389/fnut.2025.1651134 (PMC12461264; doi:10.3389/fnut.2025.1651134)
Supplement: Supplementary file 1 [file Supplementary_file_1.docx]

Supplementary Material

#

# Impact of Fermented Dairy Product Consumption on Blood Lipid Profiles in Healthy Adults: A Systematic Review

#

#

**Supplementary Table S1. Search strategy for bibliographic search of published human studies related to the health benefits and risks of dairy fermented products.**

**MEDLINE**

| No. | Query |
| --- | --- |
| #1 | (((ferment*[tiab] OR culture*[tiab] OR sour*[tiab]) AND (milk[tiab] OR dairy[tiab])) OR buttermilk[tiab] OR sour cream*[tiab] OR cheese*[tiab] OR yoghurt[tiab] OR yogurt[tiab] OR "yoghourt"[tiab] OR "yakult"[tiab] OR "quark"[tiab] OR "kefir"[tiab] OR "lassi"[tiab] OR "kumis"[tiab] OR "koumiss"[tiab] OR "kajmak"[tiab] OR "airag"[tiab] OR "ayran"[tiab] OR "calpis"[tiab] OR "borhani"[tiab] OR "chal"[tiab] OR "doogh"[tiab] OR kvass[tiab] OR skyr[tiab] OR amasi[tiab] OR bouza[tiab] OR butter*[tiab] OR chal[tiab] OR **filmjolk**[tiab] OR kishk[tiab] OR labne*[tiab] OR dahi[tiab]) |
| #2 | "Diet"[Mesh] OR "Life Style"[Mesh] OR "Eating"[Mesh] OR "Feeding Behavior"[Mesh] OR ((food[tiab] OR macronutrient*[tiab] OR eating[tiab]) AND (intake*[tiab] OR habit*[tiab] OR behavior*[tiab] OR pattern*[tiab])) OR diet*[tiab] OR intake[tiab] OR ingestion[tiab] OR suppl*[tiab] OR consumption[tiab] OR meal*[tiab] OR nutrient*[tiab] OR nutrit*[tiab] |
| #3 | ("Cardiovascular Diseases"[MeSH] OR ((cardiovascular[tiab] OR heart[tiab] OR coronary[tiab] OR cerebrovascular[tiab]) AND ("Risk Factors"[MeSH] OR factor*[tiab] OR risk*[tiab] OR rate*[tiab] OR hazard ratio*[tiab] OR disease*[tiab])) OR "CVD"[tiab] OR "myocardial infarction"[tiab:~3] OR "heart failure"[tiab:~3] OR "cardiovascular event"[tiab:~3] OR "angina"[tiab] OR "stroke"[tiab] OR "hypertension "[tiab] OR "blood pressure"[tiab:~3] OR (("blood lipids"[tiab:~3] OR "cholesterol"[tiab] OR "triglycerides"[tiab] OR "HDL"[tiab] OR "high-density lipoprotein"[tiab:~5] OR "LDL"[tiab] OR "low-density lipoprotein"[tiab:~5]) AND (level*[tiab] OR blood[tiab] OR serum[tiab] OR plasma*[tiab] OR concentration*[tiab]))) |
| #4 | #1 AND #2 AND #3 |
| #5 | "Diet Surveys"[Mesh] OR "Cohort Studies"[Mesh] OR cohort*[Tiab] OR prospective[Tiab] OR longitudinal[Tiab] |
| #6 | Randomized Controlled Trial[Publication Type] OR Controlled Clinical Trial[Publication Type] OR Pragmatic Clinical Trial[Publication Type] OR Clinical Study[Publication Type] OR Adaptive Clinical Trial[Publication Type] OR Equivalence Trial[Publication Type] OR Clinical Trial[Publication Type] OR Clinical Trial, Phase I[Publication Type] OR Clinical Trial, Phase II[Publication Type] OR Clinical Trial, Phase III[Publication Type] OR Clinical Trial, Phase IV[Publication Type] OR Clinical Trial Protocol[Publication Type] OR multicenter study[Publication Type] OR "Clinical Studies as Topic"[Mesh] OR "Clinical Trials as Topic"[Mesh] OR "Clinical Trial Protocols as Topic"[Mesh] OR "Multicenter Studies as Topic"[Mesh] OR "Random Allocation"[Mesh] OR "Double-Blind Method"[Mesh] OR "Single-Blind Method"[Mesh] OR "Placebos"[Mesh:NoExp] OR "Control Groups"[Mesh] OR "Cross-Over Studies"[Mesh] OR random*[Title/Abstract] OR sham[Title/Abstract] OR placebo*[Title/Abstract] OR ((singl*[Title/Abstract] OR doubl*[Title/Abstract]) AND (blind*[Title/Abstract] OR dumm*[Title/Abstract] OR mask*[Title/Abstract])) OR ((tripl*[Title/Abstract] OR trebl*[Title/Abstract]) AND (blind*[Title/Abstract] OR dumm*[Title/Abstract] OR mask*[Title/Abstract])) OR "control study"[tiab:~3] OR "control studies"[tiab:~3] OR "control group"[tiab:~3] OR "control groups"[tiab:~3] OR "healthy volunteers"[tiab:~3] OR "control trial"[tiab:~3] OR "control trials"[tiab:~3] OR "controlled study"[tiab:~3] OR "controlled trial"[tiab:~3] OR "controlled studies"[tiab:~3] OR "controlled trials"[tiab:~3] OR "clinical study"[tiab:~3] OR "clinical studies"[tiab:~3] OR "clinical trial"[tiab:~3] OR "clinical trials"[tiab:~3] OR Nonrandom*[Title/Abstract] OR non random*[Title/Abstract] OR non-random*[Title/Abstract] OR quasi-random*[Title/Abstract] OR quasirandom*[Title/Abstract] OR "phase study"[tiab:~3] OR "phase studies"[tiab:~3] OR "phase trial"[tiab:~3] OR "phase trials"[tiab:~3] OR "crossover study"[tiab:~3] OR "crossover studies"[tiab:~3] OR "crossover trial"[tiab:~3] OR "crossover trials"[tiab:~3] OR "cross-over study"[tiab:~3] OR "cross-over studies"[tiab:~3] OR "cross-over trial"[tiab:~3] OR "cross-over trials"[tiab:~3] OR ((multicent*[tiab] OR multi-cent*[tiab] OR open label[tiab] OR open-label[tiab] OR equivalence[tiab] OR superiority[tiab] OR non-inferiority[tiab] OR noninferiority[tiab] OR quasiexperimental[tiab] OR quasi-experimental[tiab]) AND (study[tiab] OR studies[tiab] OR trial*[tiab])) OR allocated[tiab] OR pragmatic study[tiab] OR pragmatic studies[tiab] OR pragmatic trial*[tiab] OR practical trial*[tiab] |
| #7 | "Epidemiologic Methods"[Mesh:NoExp] OR "Epidemiologic Studies"[Mesh] OR "Observational Studies as Topic"[Mesh] OR "Clinical Studies as Topic"[Mesh] OR "Single-Case Studies as Topic"[Mesh] OR "Organizational Case Studies"[Mesh] OR observational study[Publication Type] OR validation study[Publication Type] OR clinical study[Publication Type] OR case reports[Publication Type] OR "observational study"[tiab:~3] OR "observational studies"[tiab:~3] OR "observational design"[tiab:~3] OR "observational analysis"[tiab:~3] OR "observational analyses"[tiab:~3] OR ((cohort*[tiab] OR prospective[tiab] OR follow-up[tiab] OR longitudinal[tiab] OR long-term[tiab] OR retrospective[tiab]) AND (study[tiab] OR studies[tiab] OR design[tiab] OR analysis[tiab] OR analyses[tiab] OR data[tiab] OR review[tiab])) OR case control*[tiab] OR case comparison*[tiab] OR case-referent[tiab] OR "population study"[tiab:~3] OR "population studies"[tiab:~3] OR "population analysis"[tiab:~3] OR "population analyses"[tiab:~3] OR "descriptive study"[tiab:~3] OR "descriptive studies"[tiab:~3] OR "descriptive design"[tiab:~3] OR "descriptive analysis"[tiab:~3] OR "descriptive analyses"[tiab:~3] OR "multidimensional study"[tiab:~3] OR "multidimensional studies"[tiab:~3] OR "multidimensional design"[tiab:~3] OR "multidimensional analysis"[tiab:~3] OR "multidimensional analyses"[tiab:~3] OR "cross-sectional study"[tiab:~3] OR "cross-sectional studies"[tiab:~3] OR "cross-sectional design"[tiab:~3] OR "cross-sectional analysis"[tiab:~3] OR "cross-sectional analyses"[tiab:~3] OR "cross-sectional research"[tiab:~3] OR "cross-sectional survey"[tiab:~3] OR "cross-sectional findings"[tiab:~3] OR natural experiment*[tiab] OR quasi experiment*[tiab] OR "nonexperimental study"[tiab:~3] OR "nonexperimental studies"[tiab:~3] OR "nonexperimental design"[tiab:~3] OR "nonexperimental analysis"[tiab:~3] OR "nonexperimental analyses"[tiab:~3] OR "prevalence study"[tiab:~3] OR "prevalence studies"[tiab:~3] OR "prevalence analysis"[tiab:~3] OR "prevalence analyses"[tiab:~3] OR case series[tiab] OR "case report"[tiab:~3] OR "case reports"[tiab:~3] OR "case study"[tiab:~3] OR "case studies"[tiab:~3] OR "case histories"[tiab:~3] |
| #8 | "systematic review" |
| #9 | #5 OR #6 OR #7 OR #8 |
| #10 | #4 AND #9 |
| #11 | #10 NOT (("Child"[Mesh] OR "Infant"[Mesh] OR "Adolescent"[Mesh]) NOT "Adult"[Mesh]) |
| #12 | #11 NOT (("Animals"[Mesh] OR "Animal Experimentation"[Mesh] OR "Models, Animal"[Mesh] OR "Vertebrates"[Mesh]) NOT ("Humans"[Mesh] OR "Human Experimentation"[Mesh])) |
| #13 | #12 NOT ("Breast Feeding"[Majr] OR "Milk, Human"[Majr]) |
| #14 | #13 AND (English[Filter]) |
| #15 | #14 AND (("1970/01/01"[Date - Publication] : "2023/08/31"[Date - Publication])) |

**SCOPUS**

| # | Query |
| --- | --- |
| #1 | TITLE-ABS-KEY (((ferment* OR culture* OR sour*) AND (milk OR dairy)) OR buttermilk OR sour cream* OR cheese* OR yoghurt OR yogurt OR yoghourt OR yakult OR quark OR kefir OR lassi OR kumis OR koumiss OR kajmak OR airag OR ayran OR calpis OR borhani OR chal OR doogh OR skyr OR amasi OR bouza OR butter* OR chal OR filmjolk OR kishk OR labne* OR dahi) |
| #2 | TITLE-ABS-KEY (((food OR *nutrient* OR eating OR nutrit*) W/6 (intake* OR habit* OR behavior* OR pattern* OR consumption OR suppl* OR ingestion)) OR diet* OR meal*) |
| #3 | TITLE-ABS (("Cardiovascular Diseases") OR ((cardiovascular OR heart OR coronary OR cerebrovascular) AND (("Risk Factors") OR factor* OR risk* OR rate* OR ("hazard ratio*") OR disease*)) OR CVD OR (myocardial W/3 infarction) OR (heart W/3 failure) OR (cardiovascular W/3 event) OR angina OR stroke OR hypertension OR (blood W/3 pressure) OR (((blood W/3 lipids) OR cholesterol OR triglycerides OR HDL OR (high-density W/5 lipoprotein) OR LDL OR (low-density W/5 lipoprotein)) AND (level* OR blood OR serum OR plasma* OR concentration*))) |
| #4 | #1 AND #2 AND #3 |
| #5 | TITLE-ABS-KEY (random* OR sham OR placebo*) OR TITLE-ABS-KEY ((singl* OR doubl*) W/1 (blind* OR dumm* OR mask*)) OR TITLE-ABS-KEY ((tripl* OR trebl*) W/1 (blind* OR dumm* OR mask*)) OR TITLE-ABS-KEY (control* W/3 (study OR studies OR trial* OR group*)) OR TITLE-ABS-KEY (clinical W/3 (study OR studies OR trial*)) OR TITLE-ABS-KEY (Nonrandom* OR "non random*" OR non-random* OR quasi-random* OR quasirandom*) OR TITLE-ABS-KEY (phase W/3 (study OR studies OR trial*)) OR TITLE-ABS-KEY ((crossover OR cross-over) W/3 (study OR studies OR trial*)) OR TITLE-ABS-KEY ((multicent* OR multi-cent*) W/3 (study OR studies OR trial*)) OR TITLE-ABS (allocated) OR TITLE-ABS-KEY (("open label" OR open-label) W/5 (study OR studies OR trial*)) OR TITLE-ABS-KEY ((equivalence OR superiority OR non-inferiority OR noninferiority) W/3 (study OR studies OR trial*)) OR TITLE-ABS-KEY ("pragmatic study" OR "pragmatic studies") OR TITLE-ABS-KEY ((pragmatic OR practical) W/3 trial*) OR TITLE-ABS-KEY ((quasiexperimental OR quasi-experimental) W/3 (study OR studies OR trial*)) OR TITLE (trial) OR KEY (trial) |
| #6 | TITLE-ABS-KEY (observational W/3 (study OR studies OR design OR analysis OR analyses)) OR TITLE-ABS-KEY (cohort*) OR TITLE-ABS-KEY (prospective W/7 (study OR studies OR design OR analysis OR analyses)) OR TITLE-ABS-KEY (("follow up" OR followup) W/7 (study OR studies OR design OR analysis OR analyses)) OR TITLE-ABS-KEY ((longitudinal OR longterm OR (long W/1 term)) W/7 (study OR studies OR design OR analysis OR analyses OR data)) OR TITLE-ABS-KEY (retrospective W/7 (study OR studies OR design OR analysis OR analyses OR data OR review)) OR TITLE-ABS-KEY ((case W/1 control) OR (case W/1 comparison) OR (case W/1 controlled)) OR TITLE-ABS-KEY (case-referent W/3 (study OR studies OR design OR analysis OR analyses)) OR TITLE-ABS-KEY (population W/3 (study OR studies OR analysis OR analyses)) OR TITLE-ABS-KEY (descriptive W/3 (study OR studies OR design OR analysis OR analyses)) OR TITLE-ABS-KEY ((multidimensional OR (multi W/1 dimensional)) W/3 (study OR studies OR design OR analysis OR analyses)) OR TITLE-ABS-KEY (cross W/1 sectional W/7 (study OR studies OR design OR research OR analysis OR analyses OR survey OR findings)) OR TITLE-ABS-KEY ((natural W/1 experiment) OR (natural W/1 experiments)) OR TITLE-ABS-KEY (quasi W/1 (experiment OR experiments OR experimental)) OR TITLE-ABS-KEY (("non experiment" OR nonexperiment OR "non experimental" OR nonexperimental) W/3 (study OR studies OR design OR analysis OR analyses)) OR TITLE-ABS-KEY (prevalence W/3 (study OR studies OR analysis OR analyses)) OR TITLE-ABS-KEY ("case series") OR TITLE-ABS-KEY (case W/3 (report OR reports OR study OR studies OR histories)) |
| #7 | TITLE-ABS-KEY ("systematic review") |
| #8 | #5 OR #6 OR #7 *(add combination of string numbers to the field in advanced search in the field "Combined queries…")* |
| #9 | #4 AND #8 *(add combination of string numbers to the field in advanced search in the field "Combined queries…")* |
| #10 | (KEY (animal* OR nonhuman)) AND NOT (KEY (human*)) |
| #11 | #9 AND NOT #10  *(add combination of string numbers to the field in advanced search in the field "Combined queries…")* |
| #12 | (KEY (infant* OR child*)) AND NOT (KEY (adult* OR aged)) |
| #13 | #11 AND NOT #12  *(add combination of string numbers to the field in advanced search in the field "Combined queries…")* |
|  | *Limit #13 to English using the language filter* |
|  | *Limit #13 to 1970 - 2023 using the Year filter (Range from 1970 to 2023)* |

**COCHRANE**

| ID | Search in ti,ab,kw |
| --- | --- |
| #1* | (((ferment*:ti,ab OR culture*:ti,ab OR sour*:ti,ab) AND (milk:ti,ab OR dairy:ti,ab)) OR buttermilk:ti,ab OR ("sour" NEXT cream*):ti,ab OR cheese*:ti,ab OR yoghurt:ti,ab OR yogurt:ti,ab OR yoghourt:ti,ab OR yakult:ti,ab OR quark:ti,ab OR kefir:ti,ab OR lassi:ti,ab OR kumis:ti,ab OR koumiss:ti,ab OR kajmak:ti,ab OR airag:ti,ab OR ayran:ti,ab OR calpis:ti,ab OR borhani:ti,ab OR chal:ti,ab OR doogh:ti,ab OR kvass:ti,ab OR skyr:ti,ab OR amasi:ti,ab OR bouza:ti,ab OR butter*:ti,ab OR chal:ti,ab OR filmjolk:ti,ab OR kishk:ti,ab OR labne*:ti,ab OR dahi:ti,ab) |
| #2* | (([mh Diet] OR [mh "Life Style"] OR [mh Eating] OR [mh "Feeding Behavior"]):ti,ab OR ((food:ti,ab OR macronutrient*:ti,ab OR eating:ti,ab) AND (intake*:ti,ab OR habit*:ti,ab OR behavior*:ti,ab OR pattern*:ti,ab)) OR diet*:ti,ab OR intake:ti,ab OR ingestion:ti,ab OR suppl*:ti,ab OR consumption:ti,ab OR meal*:ti,ab OR nutrient*:ti,ab OR nutrit*:ti,ab) |
| #3* | (([mh "Cardiovascular Diseases"]:ti,ab) OR ((cardiovascular:ti,ab OR heart:ti,ab OR coronary:ti,ab OR cerebrovascular:ti,ab) AND ([mh "Risk Factors"]:ti,ab OR factor*:ti,ab OR risk*:ti,ab OR rate*:ti,ab OR ("hazard" NEXT ratio*):ti,ab OR disease*:ti,ab)) OR CVD:ti,ab OR (myocardial NEAR/3 infarction) OR (heart NEAR/3 failure) OR (cardiovascular NEAR/3 event) OR angina:ti,ab OR stroke:ti,ab OR hypertension:ti,ab OR (blood NEAR/3 pressure) OR (((blood NEAR/3 lipids) OR cholesterol:ti,ab OR triglycerides:ti,ab OR HDL:ti,ab OR (high-density NEAR/5 lipoprotein):ti,ab OR LDL:ti,ab OR (low-density NEAR/5 lipoprotein):ti,ab) AND (level*:ti,ab OR blood:ti,ab OR serum:ti,ab OR plasma*:ti,ab OR concentration*:ti,ab))) |
| #4* | #1 AND #2 AND #3 |
| ** | Choose in the field "Limits" the following options: (a) Content type: "Cochrane Reviews" and "Trials"; (b) Date published on the Cochrane Library: Between "January 1970" and "August 2023" |

# Supplementary Table 2: The data extraction form used in the review

| **Data extraction field name** | **Short description** | **Field to fill** |
| --- | --- | --- |
| **General Information** |  |  |
| Date DE form completed | Use format dd.mm.yyyy |  |
| Name of person extracting data | Use your full name |  |
| **Study Identification Information** |  |  |
| Number of the publication on Cadima |  |  |
| Reference (doi) | Use full link format, e.g. https://doi.org/11.0000/nutrit/nuac00 |  |
| Publication title | Copy-paste publication title |  |
| **METHODS** | **Extract data as stated in report/paper. If some information is not reported in the publication, write "Not reported". Do not leave any fields blank.  If something is unclear, copy-paste the relevant information and comment on what is unclear.** |  |
| Aim/objective of study | Copy the sentence in the Abstract or Introduction containing the aim/objective of the study. Choose the most complete version. |  |
| Study design | e.g. Parallel RCT, crossover RCT, cluster RCT, cohort study, case-control study, cross-sectional study, other study design. Please describe any run-in or washout period. |  |
| Start date | Use the most complete option available. Use format dd.mm.yyyy, mm.yyyy OR yyyy |  |
| End date | Use the most complete option available. Use format dd.mm.yyyy, mm.yyyy OR yyyy |  |
| Duration of participation | From recruitment to last follow-up. Day, weeks, months etc. As stated in paper. Please do not convert and add time unit. |  |
| **PARTICIPANTS** | **Extract data as stated in report/paper.  Include comparative information for each intervention or comparison Group if available. Aspects should be extracted are those that could affect presence or magnitude of an effect and could help review users assess applicability to populations beyond the review. If some information is not reported in the publication, write "Not reported". Do not leave any fields blank.  If something is unclear, copy-paste the relevant information and comment on what is unclear.** |  |
| Country/region |  |  |
| Population description | E.g., healthy, specific health conditions (e.g., diabetes, hypertension etc.), comorbidities etc. |  |
| Inclusion criteria |  |  |
| Exclusion criteria |  |  |
| Total number randomised for RCT |  |  |
| **Intervention 1:** No. randomised |  |  |
| **Intervention 1:** mean/median age with variance | E.g., 67.5 ± 5.1 (mean, SD) OR 65 [60, 75] (median [IQR] |  |
| **Intervention 1:** No. F/ n M | E.g., 15/20 |  |
| **Comparison:** No. randomised |  |  |
| **Comparison:** mean/median age with variance | E.g., 67.5 ± 5.1 (mean, SD) OR 65 [60, 75] (median [IQR] |  |
| **Comparison:** No. F/ n M | E.g., 15/20 |  |
| Notes | Note additional details if needed |  |
| **INTERVENTION/EXPOSURE vs COMPARISON** | **As stated in report/paper. If some information is not reported in the publication, write "Not reported". Do not leave any fields blank.  If something is unclear, copy-paste the relevant information and comment on what is unclear.** |  |
| **Intervention 1:** Description | Include sufficient detail, e.g. dose, content, components. Description of Intervention/Exposure as defined in "I" in PICO in Study Protocol |  |
| **Intervention 1:** Duration of treatment/exposure period | Day, weeks, months etc. As stated in paper. Please do not convert and add time unit. |  |
| **Intervention 1:** Timing | e.g. Frequency, duration of each episode |  |
| **Intervention 1:** Delivery | For intervention studies, e.g. standardised meal or similar |  |
| **Comparison:** Description | Include sufficient detail, e.g. dose, content, components. Description of Comparator as defined in "C" in PICO in Study Protocol. |  |
| **Comparison:** Duration of treatment/exposure period | Day, weeks, months etc. As stated in paper. Please do not convert and add time unit. |  |
| **Comparison:** Timing | e.g. Frequency, duration of each episode |  |
| **Comparison:** Delivery | For intervention studies, e.g. standardised meal or similar |  |
| Notes | Note additional details (i.e reported adverse effects) if needed |  |
| **OUTCOMES - DATA AND ANALYSIS** | **As stated in report/paper. If some information is not reported in the publication, write "Not reported". Do not leave any fields blank.  If something is unclear, copy-paste the relevant information and comment on what is unclear. Use the appropriate section depending on the type of Outcome reported in the publication (Dichotomous, Continuous, or Other).** |  |
| **Primary Outcomes (extend this section as needed)** |  |  |
| Outcome definition | With diagnostic criteria if relevant |  |
| Method used for measurement | Measurement tool, instrument, name of the scale |  |
| Unit of measurement | E.g., mg/dL, mmol/L etc. (Mandatory: please do not perform conversions) |  |
| Mean unit | E.g., mean, median etc. (Mandatory: please do not perform conversions) |  |
| Variance unit | E.g., standard deviation, stand error etc. (Mandatory: please do not perform conversions) |  |
| ***INTERVENTION 1*** *Baseline* |  |  |
| **Intervention:** No. Participant with results |  |  |
| **Intervention:** Mean | E.g., 137 |  |
| **Intervention:** Variance | E.g., 25 |  |
| ***INTERVENTION 1*** *Endline* |  |  |
| **Intervention:** No. Participant with results |  |  |
| **Intervention:** Mean | E.g., 137 |  |
| **Intervention:** Variance | E.g., 25 |  |
| ***COMPARISON*** *Baseline* |  |  |
| **Comparison:** No. Participant with results |  |  |
| **Comparison:** Mean | E.g., 132 |  |
| **Comparison:** Variance | E.g., 19 |  |
| ***COMPARISON*** *Endline* |  |  |
| **Comparison:** No. Participant with results |  |  |
| **Comparison:** Mean | E.g., 132 |  |
| **Comparison:** Variance | E.g., 19 |  |
| Paper conclusion about the outcome | Copy and paste from the paper. E.g., "No significant difference between I and C groups", "TAG significantly reduced in the I compared to the C group". |  |
| Notes | Note additional details if needed |  |
| **Secondary Outcomes (extend this section as needed)** |  |  |
| Is it mentioned in the study? | Yes/No |  |
| Outcome definition | With diagnostic criteria if relevant |  |
| Method used for measurement | Measurement tool, instrument, name of the scale |  |
| Unit of measurement | E.g., mg/dL, mmol/L etc. (Mandatory: please do not perform conversions) |  |
| Mean unit | E.g., mean, median etc. (Mandatory: please do not perform conversions) |  |
| Variance unit | E.g., standard deviation, stand error etc. (Mandatory: please do not perform conversions) |  |
| ***INTERVENTION 1*** *Baseline* |  |  |
| **Intervention:** No. Participant with results |  |  |
| **Intervention:** Mean | E.g., 137 |  |
| **Intervention:** Variance | E.g., 25 |  |
| ***INTERVENTION 1*** *Endline* |  |  |
| **Intervention:** No. Participant with results |  |  |
| **Intervention:** Mean | E.g., 137 |  |
| **Intervention:** Variance | E.g., 25 |  |
| ***COMPARISON*** *Baseline* |  |  |
| **Comparison:** No. Participant with results |  |  |
| **Comparison:** Mean | E.g., 132 |  |
| **Comparison:** Variance | E.g., 19 |  |
| ***COMPARISON*** *Endline* |  |  |
| **Comparison:** No. Participant with results |  |  |
| **Comparison:** Mean | E.g., 132 |  |
| **Comparison:** Variance | E.g., 19 |  |
| Paper conclusion about the outcome | Copy and paste from the paper. E.g., "No significant difference between I and C groups", "TAG significantly reduced in the I compared to the C group". |  |
| Notes | Note additional details if needed |  |
| **OTHERS** |  |  |
| Compliance | This can be reported differently across studies. Please add any information found. Copy and paste from paper |  |
| Dropout rate | Give No. and reasons mentioned for dropouts. Commonly found on **CONSORT** flowchart. |  |
| Diet information | Was diet assessed at baseline and post-intervention? Was there any change in general or difference between groups that could influence the outcomes? |  |
| Results retrieved from per-protocol or intention-to-treat analyses | Please extract "intention-to-treat analyses" results if available |  |

**Supplementary Table 3: Characteristics of the Observational Studies (Cross-sectional)**

| Author, year, country | Population | Dietary assessment | Exposure(s) | Frequency/amount | Covariates | Main findings |
| --- | --- | --- | --- | --- | --- | --- |
| Salinas-Mandujano RG, et al., 2023, México (66) | Healthy adults, aged 18-25 years, 184 F/ 156 M | Semi-quantitative 160-item FFQ | Tertiles of yogurt drink pattern defined using principal components analysis (main loading factors were drinkable plain yogurt, drinkable flavoured yogurt, and lactic fermented dairy drink, and explained 9.38% of the variance) | Participants reported intake frequency and portion size using visual aids, which were converted to millilitres | Sex, smoking status, physical activity, total energy intake, and % energy from protein, fat,  and carbohydrates | No differences were observed for TG, TC, LDL-c, HDL-c, BP, %BF, and WC. Highest tertile of yogurt pattern was associated with lower odds for high glucose (OR: 0.110; 95% CI: 0.22–0.559). |
| Kim J, 2013, Korea  (64) | Korean National Health and Nutrition Examination Survey (2869 F 48.5 ± 15.6 years, 1993 M 50.6 ± 15.9 years) | FFQ | Frequency of yoghurt (low-fat, skim, and whole yogurt) serving (none or rarely, ≤2–3 per month, ≤4–6 per week, ≥ once per day) |  | Age, sex, education level, income, smoking status, BMI, alcohol intake, physical activity, and intake of energy, fat (g/d), calcium, and fibre (g/day) | Higher yogurt consumption was associated with a significantly lower risk of low HDL cholesterol (OR = 0.72, 95% CI = 0.52–1.00, *P* for trend = 0.0197) and a reduced overall risk of metabolic syndrome (OR = 0.71, 95% CI = 0.48–1.05, *P* for trend = 0.0067). |
| Shi N, 2021, United States (63) | 35,352,  postmenopausal women | FFQ | Cheese and yogurt | servings per day |  | Higher yogurt intake was associated with lower total cholesterol (−1.1%) and higher HDL cholesterol (+1.8%). Overall, greater dairy consumption, particularly yogurt and cheese, was linked to lower levels of triglycerides, glucose, insulin, and C-reactive protein. |
| Wang H, 2013, United States (65) | 6526 | FFQ | Yogurt | yogurt consumers (>0 servings/week) vs no consumers (0 servings/week). |  | Yogurt consumption was linked to lower levels of circulating triglycerides, glucose, systolic blood pressure, and insulin resistance (all p < 0.05). |
| Feeney E, 2017, Ireland (46) | 760 F, 740 M | 4-day food diary | Cheese and yogurt | Based on consuming amount |  | Higher yogurt intake was associated with significantly lower body fat, waist circumference, and waist-to-hip ratio (p< 0.05), as well as reduced TNF-α levels (p< 0.001).  Cheese consumption showed no consistent metabolic benefits, with only C-peptide levels increasing with higher intake (p= 0.001). |

FFQ: Food frequency questionnaire

**Supplementary Table 4: Characteristics of the Observational Studies (Prospective Cohorts)**

| Author, year, country | Cohort name | Sex and Age | Mean Follow-up (year) | Dietary assessment | Product(s) | Main findings |
| --- | --- | --- | --- | --- | --- | --- |
| Key TJ, 2019, Denmark, Germany, Greece, Italy, New Zealand, Sweden, United Kingdom, France (59) | Pan-European EPIC Study | 303134 F/106751 M  51.3 ±9.8 F / 52.7 ± 10.3 M | 12.6 | validated questionnaires and calibrated with 24-hour recalls | Yogurt and (increment 100g/day) and cheese (increment 30g/day) | Higher yogurt intake was associated with a reduced risk of ischemic heart disease (HR: 0.93 per 100 g/day; 95% CI: 0.89–0.98), though this association became non-significant after excluding the first 4 years of follow-up, suggesting potential reverse causation. Yogurt intake was also linked to lower non-HDL cholesterol, a possible mediator of cardiovascular benefits. |
| Guo J, 2022, Denmark (53) | MONICA-I cohort (1982-1984) | 1746 (52% F), 30-60 years | 30 | 7-day weighed diet diary | Total fermented dairy (quartiles) | No significant associations were found for dairy subtypes, including yogurt specifically. |
| Silva FM, 2021, Brazil (55) | ELSA-Brasil | 6671 (50.1% F), 52±9 years | 8 | FFQ | Sex-specific quartiles of consumption adjusted for calories (g/d) | No associations were found between low-fat or fermented dairy and cardiovascular mortality. |
| Sellem L, 2021, France (54) | NutriNet-Santé | 104805,  42.8 ± 14.6 years | 5.5±3.0 | 24-h dietary records (3 days) | Fermented dairy products (increment of 30g/d of cheese, and 100 g/d of yogurt) | No significant links between total dairy intake and risks of total CVD or CHD.  Consuming at least 160 g/day of fermented dairy (cheese and yogurts) was associated with a 19% lower risk of cerebrovascular disease compared to consuming less than 57 g/day. |
| Buziau AM, 2019, Australia (10) | Australian Longitudinal Study on Women’s Health | 7633 F, 45-50 years | 5 | FFQ | Yogurt, cheese, total fermented dairy | Higher intake of yogurt and total fermented dairy was linked to lower CVD risk, but these associations weakened after further adjustments. |
| Johansson I, 2019, Sweden (62) | Västerbotten Intervention Programme (VIP) and the Northern Sweden | 108065 | 14.2 | FFQ | Fermented milk, cheese | No significant association between dairy consumption and the risk of stroke. |
| Dalmeijer GW, 2013, The Netherlands (61) | EPIC-NL | 33625 (74.5% F), 49±11.9 years | 13.1 | FFQ | Fermented dairy | No dairy subtypes were significantly linked to CHD risk, though fermented dairy showed a borderline trend toward reduced stroke risk (HR = 0.92; 95% CI: 0.83–1.01; p = 0.07). |
| Kouvari M, 2020, Greece (57) | ATTICA study | 1528 F / 1514 M  F: 45±14, M:46±13 years | 8.41 | FFQ | Yogurt 200 g/day, cheese 30g/day | Each 200 g/day increase in yogurt consumption was associated with 20-30% lower CVD risk, especially in women.  Each 30 g/day increase in cheese intake was associated with ~5% lower CVD risk, particularly in men. |
| Zhang S, 2023, Sweden (12) | Swedish Malmö Diet and Cancer  Study (MDCS) cohort | 20499 (61.6% F), 57.8±7.6 years | 21 | 7-day food diary and FFQ | fermented milk (yogurt and sour milk). | Fermented milk appears to be protective against CVD and related mortality. |
| Lu Y, 2021, Japan (60) | Miyagi Cohort Study | 17596 F / 16565 M, 40-64 years | 25 | FFQ | Yogurt and cheese | Yogurt intake: No significant associations with any type of mortality. Cheese: Showed a modest inverse association with all-cause mortality in women. |
| Koskinen TT, 2018, Finland (9) | Kuopio Ischaemic Heart Disease Risk Factor Study | 1981 M, 42-60 years | 20.1 | 4-d food records | Fermented dairy products | Fermented dairy, especially low-fat, is associated with 27% lower CHD risk.  Non-fermented dairy is associated with increased CHD risk. |
| Patterson E, 2012, Sweden (56) | Swedish Mammography Cohort | 33636 F, 48-83 years | 11.6 | FFQ | Cultured milk/yogurt, chee | Cheese: Inversely associated with MI risk, HR = 0.74 (95% CI: 0.60–0.91). Cultured milk/yogurt: No significant associations with MI risk. |

CVD: Cardiovascular disease; CHD: Coronary heart disease; FFQ: Food frequency questionnaire, MI: Myocardial infarction
